# Characterization of the cell wall of a mushroom forming fungus at atomic resolution using solid-state NMR spectroscopy

**DOI:** 10.1016/j.tcsw.2020.100046

**Published:** 2020-10-28

**Authors:** Helena Leona Ehren, Freek V.W. Appels, Klaartje Houben, Marie A.M. Renault, Han A.B. Wösten, Marc Baldus

**Affiliations:** a NMR Spectroscopy, Bijvoet Center for Biomolecular Research, Utrecht University, Padualaan 8, 3584 CH Utrecht, the Netherlands; b Microbiology, Department of Biology, Utrecht University, Padualaan 8, 3584 CH Utrecht, the Netherlands

**Keywords:** Schizophyllum commune, Solid-state NMR, Fungus, Basidiomycete, Cell wall, Cell wall composition

## Abstract

Cell walls are essential in the interaction of fungi with the (a)biotic environment and are also key to hyphal morphogenesis and mechanical strength. Here, we used solid-state NMR (ssNMR) spectroscopy combined with HPLC and GC–MS to study the structural organization of the cell wall of a representative of the Basidiomycota, one of the two main phyla of fungi. Based on the data we propose a refined model for the cell wall of a basidiomycete. In this model, the rigid core is built from α- and β-(1,3)-glucan, β-(1,3)-(1,6)-glucan, highly branched and single stranded β-(1,4)-chitin as well as polymeric fucose. The mobile fraction of the cell wall is composed of β-(1,3)-glucan, β-(1,3)-(1,6)-glucan, β-(1,6)-glucan, α-linked reducing and non-reducing ends and polymeric mannose. Together, these findings provide novel insights into the structural organization of the cell wall of the model basidiomycete *S. commune* that was previously based on destructive chemical and enzymatic analysis.

## 1 Introduction

The fungal kingdom is diverse with 120.000 identified species and a total predicted number of 3.8 million representatives (Hawksworth and Lücking, 2017). Its two main phyla are the Ascomycota and the Basidiomycota that diverged 500 million years ago (Hibbett et al., 2007). Their representatives degrade dead organic matter and/or establish pathogenic or mutual beneficial interactions with plants, animals and microbes. For instance, the mushroom forming fungus *Schizophyllum commune* feeds on fallen branches and timber of deciduous trees and on softwood and grass silage but it can also be an opportunistic pathogen of plants and animals (Ohm et al., 2010).

Cell walls play essential functions in the interaction of fungi with their (a)biotic environment and are key to hyphal morphogenesis and mechanical strength (Gow et al., 2017). As such, they are of significant interest for the medical, agricultural and biotechnological field as well as for the production of sustainable materials (Gow et al., 2017, Wösten, 2019). Despite their importance, relatively little is known about fungal cell walls. Previous work has shown that the composition of fungal cell walls is dynamic and varies between species, strains, environmental conditions, and developmental stage (Bowman and Free, 2006, Erwig and Gow, 2016). Until recently, the biochemical analysis of fungal cell walls was based on destructive methods using enzymatic and/or chemical treatments. Phosphate buffered saline (PBS) is normally used for gentle washing of cells prior to, e.g., DNA or protein extraction (Andrews and Faller, 1991). More stringent solubilization extraction procedures include SDS (Klis et al., 2001) and/or alkali treatment (Sietsma and Wessels, 1977, van Wetter et al., 2000). While these approaches have offered valuable insight into the chemical composition of cell walls, such procedures provide only limited information regarding the molecular architecture of intact cell walls.

Nuclear magnetic Resonance (NMR) spectroscopy exploits the magnetic properties of nuclear spins to probe molecular structure and dynamics. In particular, the NMR resonance frequency of a nuclear spin is sensitive to the local chemical environment. In addition, through-space dipolar interactions between NMR spins are modulated by molecular motion in contrast to through-bond (a.k.a. scalar) spin–spin interactions that only depend on bonding and molecular structure. In recent years, solid-state NMR (ssNMR) has made important progress to obtain atomic-level insight into molecular structure and dynamics of complex biomolecular systems including those of cell walls of bacteria (Renault et al., 2012), plants (Serra et al., 2012, Dupree et al., 2015, Wang et al., 2016) and diatoms (Jantschke et al., 2015). In addition, the intact cell wall structure of the human pathogenic ascomycete *Aspergillus fumigatus* was studied at atomic resolution (Kang et al., 2018), indicating that α-(1,3)-glucan has a more important function than previously assumed. It was found to form a dynamic layer together with glycoproteins at the outer part of the cell wall, capping a hydrated matrix of (1,3)-, (1,4)-, and (1,6)-linked β-glucans. In turn, this matrix covers the inner layer of the cell wall consisting of a hydrophobic and rigid scaffold composed of chitin and α-(1,3)-glucan. In contrast, studies in the past indicated that chitin, β-(1,3)-glucan and β-(1,6)-glucan form the rigid part of the *A. fumigatus* cell wall (Bowman and Free, 2006).

Here, we used solid-state NMR to investigate the cell wall of the model species for mushroom formation *S. commune* (Ohm et al., 2010). Comparing ssNMR data of intact preparations with samples obtained after destructive treatments refined our results and allowed us to evaluate previous studies that used enzymatic and chemical treatments. The latter studies had revealed that the cell walls of vegetative hyphae of the basidiomycete *S. commune* consist of glucose (67.6%), N-acetylglucosamine (12.5%), mannose (3.4%), xylose (0.2%), amino acids (6.4%), and lipids (3.0%) (Sietsma and Wessels, 1977). Further work indicated that a water-soluble mucilage of β-(1,3)-(1,6)-glucan forms the outer layer of the *S. commune* cell wall (Sietsma and Wessels, 1977). This so called schizophyllan would cover an alkali-soluble α-(1,3)-linked glucan that is partly present in microcrystalline form. The inner layer of the cell wall would consist of randomly oriented chitin microfibrils cross-linked to and embedded in a highly branched β-(1,3)-(1,6)-glucan and would form the alkali-insoluble backbone of the cell wall (Sietsma and Wessels, 1977, van der Valk et al., 1977). We here show that the mobile phase of the cell wall not only consists of β-(1,3)-(1,6)-glucan but also of α-glucan and mannan residues. In addition, our studies reveal that the rigid part of the *S. commune* cell wall indeed consists of chitin and β-(1,3)-(1,6)-glucan, but also of α-(1,3)-glucan and polymeric fucose and mannose. Hence, our ssNMR experiments provide new insights in the structural organization of the cell wall of the model basidiomycete *S. commune*.

## 2 Materials and methods

### 2.1 Strain and culture conditions

*S. commune* wild type strain 4–39 (CBS 341.81) was grown at 30 °C for 6 days on a polycarbonate membrane (pore size 0.1 µm; Merckmillipore, Burlington, MA, USA) overlying minimal agar medium (MMA). MMA consisted per liter of 20 g ^13^C_6_-glucose (Buchem, Netherlands), 0.5 MgSO_4_ (Sigma-Aldrich, St Louis, MO, USA), 1.31 g (^15^NH_4_)_2_SO_4_ (Sigma-Aldrich, St Louis, MO, USA), 0.12 mg thiamine-HCl (Sigma-Aldrich, St Louis, MO, USA), 5 mg FeCl_3_, trace elements (Whitaker, 1951) and 1.5% agar. Cultures were inoculated with mycelium from the periphery of a 7-day-old colony.

### 2.2 Sample preparation

Cultures were harvested from the polycarbonate membranes (see above) and dried at ambient temperature. The mycelium was homogenized and hydrated using ultra-pure water to improve resolution in the ssNMR spectra. Alternatively, the homogenized mycelium was washed with Phosphate Buffer Saline (PBS). Part of this material was subjected to ssNMR (see below), while the rest was washed with 1% boiling SDS. The resulting cell wall fraction was again partly used for NMR studies and the other part was subsequently incubated in 1 M KOH at 60 °C. This procedure resulted in four differently treated mycelium samples which were analyzed (for details see Methods S1).

### 2.3 High performance liquid chromatography

The Sugar analysis was performed after total hydrolysis of the fungal cell walls with sulphuric acid. Concentrations of sugar monomers were determined by high-performance liquid chromatography using elution at 60 °C with 0.6 mL min^−1^ 5 mM H_2_SO_4_ and an Aminex HPX-87H column (Biorad) connected to a Waters Alliance e2695 HPLC. Peaks were detected by a refractive-index detector (Waters 2414) and a dual-wavelength absorbance detector (Waters 2489) at 210 nm and 270 nm. Raw data was processed into PeakArea’s and concentrations were determined using Empower2 software from Waters (for details see Methods S2).

### 2.4 Glycosyl linkage analysis

Permethylated alditol acetates (PMAA) were prepared according to Black et al., (Black et al., 2019). Samples (3 mg) were dissolved in dimethylsulfoxide (DMSO) and permethylated under basic conditions using iodomethane. Hydrolysis of the permethylated polysaccharides was achieved by incubation in 4 M HCl at 100 °C for several hours. Subsequently open-form monosaccharides were reduced using NaBD_4_. PMAA were obtained by acetylation of the residual free hydroxyl groups using acetic anhydride under basic conditions. The resulting PMAA mixture was injected into the GC–MS and analysed (for details see Methods S3).

### 2.5 Solid-State NMR

*S. commune* was grown on a PC membrane in medium containing ^13^C labelled glucose and ^15^N labelled (NH4)_2_SO_4_ and treated as described above. All ssNMR experiments were carried out on a 700 MHz Bruker Avance III or an 800 MHz Bruker Biospin spectrometer. Rigid molecules were probed using dipolar-based transfer schemes by combining cross-polarization (CP) with PARIS (Weingarth et al., 2010) or SPC5 (Hohwy et al., 1999) recoupling schemes. Two-dimensional dipolar-based (NCA-type (Baldus, 2002)) correlation experiments involved SPECIFIC-CP (Baldus et al., 1998) to transfer polarization from ^15^N to ^13^C nuclei.

Mobile compounds were probed using J-based INEPT (Morris and Freeman, 1979) and TOBSY (Baldus and Meier, 1996)- transfers as shown elsewhere (Andronesi et al., 2005). In addition, direct excitation spectra were recorded in order to excite all carbons present in the sample irrespective of molecular motion (see, e.g. Ref. (Labokha et al., 2013)). For further details see Methods S4, for measuring conditions see Tables S1 & S2.

## 3 Results and discussion

The phylum Basidiomycota contains 16 classes, 52 orders, 177 families, 1589 genera and more than 30,000 species (Hibbett et al., 2007). Approximately 32% of the described fungal taxa thus belong to this phylum (Dai et al., 2015). The Basidiomycota includes mushroom forming fungi as well as microfungi, such as rusts, smuts and yeasts. The composition and structural organization of the cell walls of basidiomycetes have not been well studied despite their importance for the lifestyle of these fungi and their medical implications. Moreover, it has been well established that fungal cell walls contain molecules with bioactive properties. For instance, schizophyllan (Wasser, 2002) and the hydrophobin SC3 (Akanbi et al., 2013, Wösten and Scholtmeijer, 2015) of *S. commune* have been shown to exhibit anti-tumor activities. In the following, we studied the cell wall composition of the model basidiomycete using ssNMR, HPLC and GC–MS.

### 3.1 Solid-state NMR studies

**Fig. 1:**
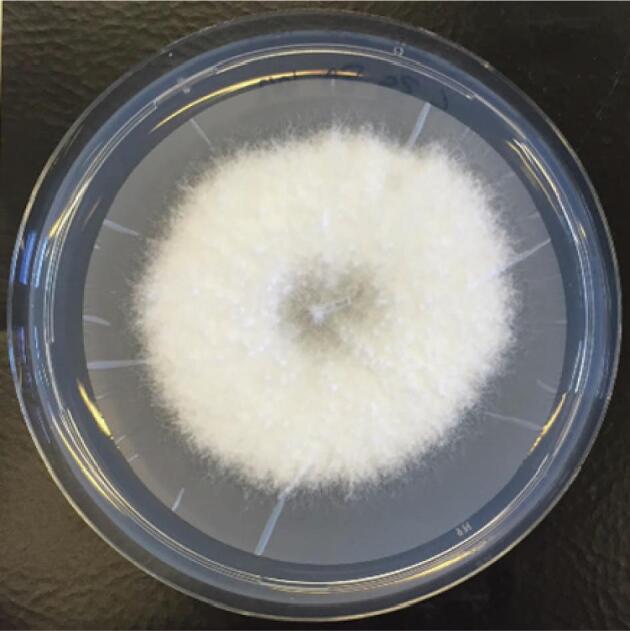
A 6-day-old *S. commune* 4–39 colony grown on a perforated polycarbonate-membrane overlaying solidified minimal medium.

**Fig. 2:**
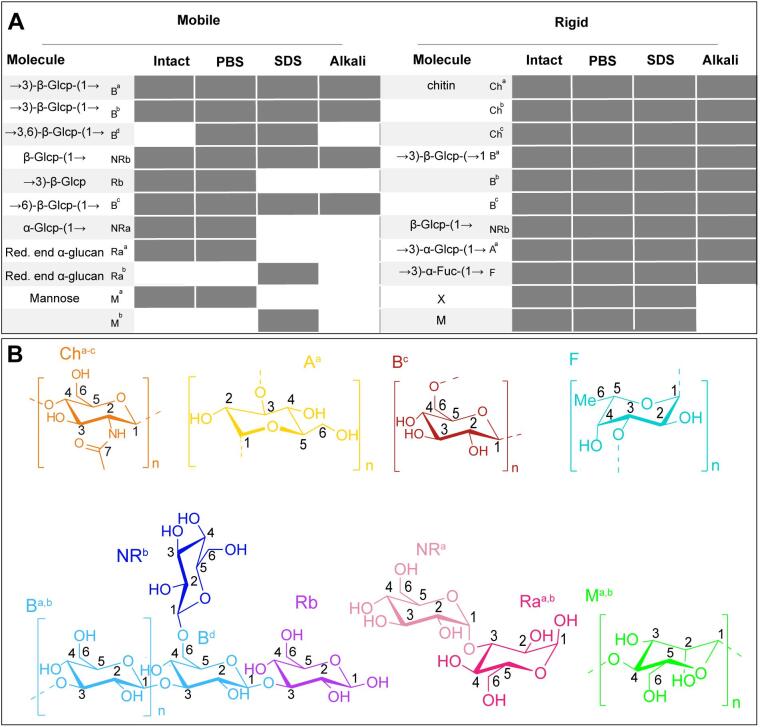
A. Flow chart of the cell wall composition for the different treatments. B. Molecular structures of identified polysaccharides and flow chart of their appearance in the samples with four different treatments.

Uniformly labeled fungal material was produced by growing mycelium for six days on a polycarbonate membrane overlying minimal medium containing ^13^C labeled glucose and ^15^N labeled (NH_4_)_2_SO_4_ (Fig. 1). We prepared four uniformly ^13^C, ^15^N-labelled samples: a) hydrated mycelium, b) PBS-washed mycelium, c) SDS-extracted mycelium and d) alkali-treated mycelium. The PBS treatment results in cytoplasm removal, while the cell wall fraction is obtained after extraction with hot-SDS. This fraction lacks hot-SDS soluble cell wall molecules including β-(1,3)-(1,6)-glucan, while the subsequent extraction with alkali has been described to remove α-(1,3)-glucan (Sietsma and Wessels, 1979, Sietsma and Wessels, 1981) and other alkali-soluble components. We then employed Magic Angle Spinning (MAS) ssNMR to study the cell wall samples. An overview of the different molecules identified in (U-^13^C,^15^N)-labeled mycelium on the basis of their ^13^C connectivities and chemical shifts known from literature (Table S3) as well as their appearance in the differently treated samples is shown in Fig. 2A. Both mobile and rigid polysaccharides were detected. Chemical shifts were indicative of β-(1,3)-(1,6)-glucan (B^a,b,d^), α-(1,3)-glucan (A^a^), β-(1,6)-glucan (B^c^) and chitin (Ch^a-c^), where the superscripts indicate the different polymorphic forms. Furthermore, fucose (F) and mannose (M^a-c^) were found. The monomeric sugar composition was confirmed by HPLC (Fig. S1). Although mannose could not be distinguished from galactose by HPLC, the experimental ssNMR chemical shifts unequivocally point to mannose as they significantly differ from known galactose shifts. Additionally, signals belonging to lipids and amino acids were observed (Fig. S2). Signals of the amino acids only appeared in the scalar-based spectra (vide infra), highlighting their mobile nature.

**Fig. 3:**
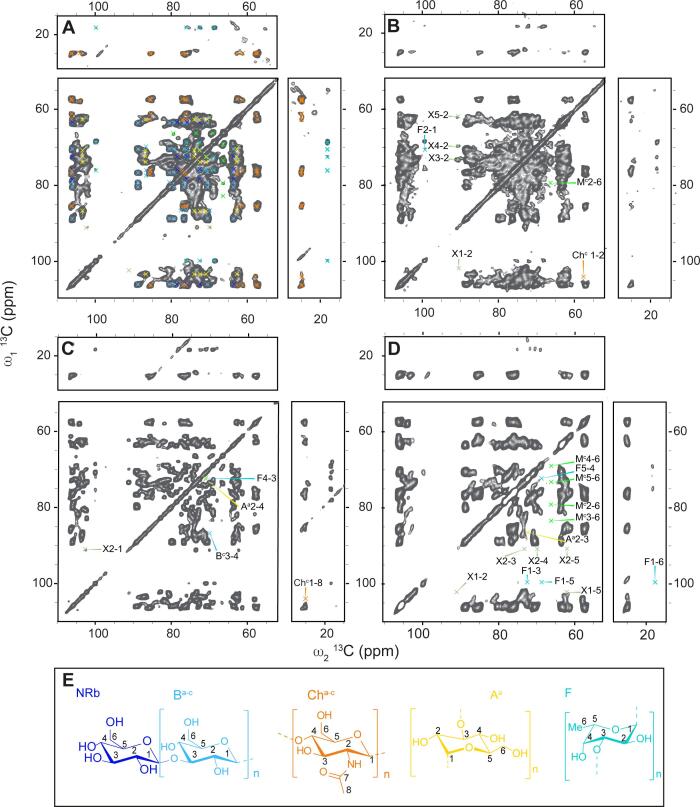
Two-dimensional ^13^C–^13^C PARIS spectra of hydrated (**A**), PBS-washed (**B**), SDS-extracted (**C**) and alkali-extracted (**D**) mycelium of *S. commune* recorded using a 120 ms mixing time showing major rigid components. Changes in the spectra arising from sequential washing steps are depicted in panels **B**, **C** and **D**. Molecular structures are shown in panel **E**. β-(1,3)-glucan (B^a-c^, light blue); non-reducing end β-(1,3)-glucan (NRb, dark blue); chitin (Ch^a-c^, orange); fucan (F, turquoise); α-(1,3)-glucan (A^a^, yellow).

Next, we focused on the identification of rigid cell wall components in the samples using ssNMR 2D sets that invoke dipolar couplings (Fig.s 3 A-D). We found that a mixing time of 120 ms under PARIS recoupling was optimal for studying carbohydrate spin systems. Chemical shifts were interpreted according to known literature shifts (Table S3) and glycosidic linkages were confirmed, when possible, for most polysaccharides by a 2D (^13^C,^13^C) double-quantum – single-quantum (DQSQ) correlation spectrum (Fig. S3) which probes one-bond connectivities between carbon atoms. Anomeric carbon atoms give rise to characteristic peaks between 99 and 102 ppm for α-linked and 103–107 ppm for β-linked polysaccharides. The glycosidic linkages at position C3 or C4 of the carbohydrate ring yielded a distinct downfield shift at 78–87 ppm. An additional connectivity between a C1 and a C6 of the adjacent sugar gave rise to a cross-peak signal of 66–70 ppm at C6. We readily could identify three different types of 1,4 linked chitin (Ch^a-c^) with the characteristic peaks for the amide carbonyl C7 between 175 and 178 ppm and the methyl group C8 between 24 and 25 ppm. The spectra also showed high intensity peaks for β-(1,3)-glucan (B^a-c^). Both, β-(1,3)-glucan and chitin are known to be part of the rigid cell wall core structure of *Schizophyllum commune*. We tentatively assigned the signals of the β-(1,3)-glucan to the backbone β-(1,3)-(1,6)-glucan. This notion is further corroborated by the presence of terminal (non-reducing end) β-linked glucose (NRb) in the spectrum. Unfortunately, it was not possible to isolate a spin system for a carbohydrate that is linked at both positions at the same time due to spectral overlap. Spectra shown in Fig.s 3A-C also revealed connectivity that would be consistent with the presence of mannose (M^c^). The observed chemical shifts of 66.0, 79.5 and 82.9 (see Table 1) indicate that mannose could be highly substituted, for example due to a ramification of another polysaccharide or a glycosylated protein. However, since we could not find cross peaks to another type of molecule, even at longer mixing times, we attribute these correlations to be part of a branched mannan. Notably, a C2 linkage in mannan is reflected by a chemical shift of C2 of around 79 ppm, a linkage at C6 often is characterized by a chemical shift of 66 ppm or higher, a linkage at C4 leads to a shift of around 80–83 ppm (Marchessault et al., 1990, Gómez-Miranda et al., 2003). Surprisingly, we also found α-(1,3)-glucan (A^a^) and fucose (F, most likely (1,3)-linked, for reference see Table S3) to be rigid. To our knowledge this has not been described before for *S. commune*. Also, for fucose we were not able to observe cross correlations to other polysaccharides, suggesting its presence either as part of fucoidan or again as a ramification. An additional carbohydrate spin-system was identified (labelled X in Fig. 3), but could not be assigned to a specific molecule.

**Table 1:** Chemical shifts of molecular moieties found within the recorded spectra based on literature values (see Table S3).

	Molecule		C1	C2	C3	C4	C5	C6	C7	C8
INEPT-TOBSY	→3)-β-Glcp-(1→	B^a^	105.0	75.3	87.3	70.5	77.9	63.1		
	→3)-β-Glcp-(1→	B^b^	105.2	75.5	86.3	70.4	78.0	63.1		
	→3,6)-β-Glcp-(1→	B^d^	103.3	72.1				68.2		
	β-Glcp-(1→	NRb	105.4	75.6	78.0	71.9	78.2	63.2		
	→3)-β-Glcp	Rb	98.4	76.8		72.1	78.4	63.2		
	→6)-β-Glcp-(1→	B^c^			78.0	71.7	77.2	71.1		
	α-Glcp-(1→	NRa	100.6	72.4		71.1				
	Reducing end α-glucan	Ra^a^	90.2	72.5		78.8				
	Reducing end α -glucan	Ra^b^	94.7	74.0		72.2	75.4	63.1		
	Mannan	M^a^		76.1	73.2	79.1	69.7	63.9		
		M^b^	102.3	74.4	73.2		69.1			
PARIS	chitin	Ch^a^	106.3	57.3	76.2	85.5	77.1	62.7	177.3	24.9
		Ch^b^	106.2	57.7	76.2	85.4	76.2	61.2	176.8	25.0
		Ch^c^	104.1	57.9					177.7	24.9
	→3)-β-Glcp-(→1	B^a^	106,0	76.7	88.7	70.5	80.5	63.6		
		B^b^	106,0	76.7	88.7	70.5	79.2	63.6		
		B^c^	106.4	76,0	86.6	69.8	77.4	63.1		
	β-Glcp-(1→	NRb	106,0	75.6	78.4	72.4	78.4	63.5		
	→3)-α-Glcp-(1→	A^a^	103.4	72.5	86.7	70.5	73.9	62.7		
	→3)-α-Fuc-(1→	F	99.8	70.5	76.0	72.5	68.7	18.2		
		X	102.4	91.0	73.3	69.8		62.2		
	branched mannose	M^c^		79.5	69.1	82.9	73.2	66.0

The ssNMR spectra obtained after different treatments of the hydrated mycelium did not reveal large differences in the cell wall composition (Fig. 3B-D). This is not surprising considering that dipolar-based ssNMR experiments reveal the most rigid core structure of the fungal cell wall that is less solvent-accessible. Washing the mycelium with PBS resulted in minor spectral changes (Fig. 3B, Table 1). Overall, cross peaks with decreased intensities were observed within the aliphatic region (10–45 ppm, see Fig. S4, S5), probably due to the loss of lipids. However, additional peaks belonging to fucose (F) and the correlations of an unidentified molecule X became visible. Hydrolysis experiments have shown the presence of xylose and some of the chemical shifts are similar to the shifts found for (1,3)-linked β-xylan in *C. taxifolia* (Lahaye et al., 2003). This would also explain the presence of only five resonances in this spin system. Extracting the PBS-washed mycelium with hot-SDS removed all protein and lipid signals (see Fig. S6), and increased the spectral resolution considerably (Fig. 3C). The lost signal belonging to X in PBS-washed mycelium was resolved again in the spectrum of SDS extracted material, whereas decreased signal intensity was found for fucose (F) and α-(1,3)-glucan (A^a^). Surprisingly, these two compounds were not removed after alkali treatment. Subsequent alkali treatment further improved the spectral resolution (Fig.s 3D, S7). Signals belonging to the unidentified molecules X and mannose were removed, while decreased signal intensity was found for fucose (F) and α-(1,3)-glucan (A^a^). Surprisingly, these two compounds were not removed after alkali treatment. Remaining signals belonged to β-(1,3)-glucan, β-(1,3)-(1,6)-glucan and chitin.

For the analysis of rigid parts of peptide or proteins, we recorded a 2D NCA spectrum (Fig. S8). This spectrum was dominated by the amide resonance of chitin and did not show signals corresponding to proteins or peptides. After extended measurements times (16 k scans), signals belonging to arginine and lysine side chains appeared in the spectra (Fig. S9). Notably, these signals did not appear in any spectra using other treatments. Interestingly, peptides have been described to covalently link polysaccharides in fungal cell walls that should be still present after SDS and alkali treatment (Sietsma and Wessels, 1977).

Taken together, we found three different polymorphic structures of chitin (Ch^a-c^) and β-(1,3)-glucan (B^a-c^), β-linked non-reducing ends (NRb), α-(1,3)-glucan (A^a^), fucose (F), mannose (M) and a yet unidentified carbohydrate (X) in the rigid part of hydrated mycelium of *S. commune.* Apart from mannose (M) and an unknown sugar (X) all the above-mentioned polysaccharides remained detectable throughout the different treatment steps confirming that they are part of a very stable, probably highly cross-connected and therefore rigid backbone structure. Mannose (M) and the unidentified molecule X were not stable towards the alkali treatment, most likely because they are less tightly associated with the core structures. Rigid parts of proteins or peptides appeared only in the intact, hydrated mycelium and with a very low abundance compared to polysaccharides (Fig. S8, Fig S9).

**Fig. 4:**
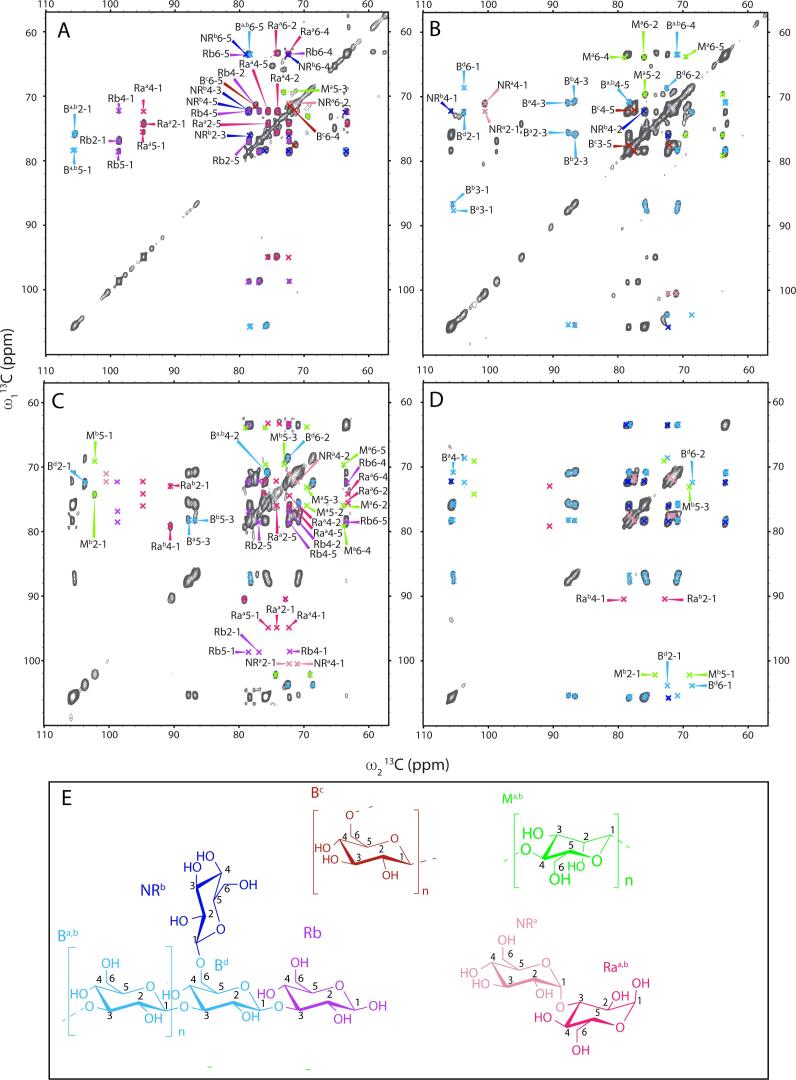
Two-dimensional (^13^C–^13^C) INEPT-TOBSY spectra obtained on hydrated (**A**), PBS-washed (**B**), SDS-extracted (**C**) and alkali-extracted (**D**) mycelium of *S. commune*. Signals appearing as a result of the washing step are annotated on the left of the diagonal in **B**, **C** and **D**. Signals disappearing after the washing procedure are annotated on the right of the diagonal in the spectra. All chemical shifts belonging to the specific molecule are given in Table 1. Molecular structure of molecules are shown in panel **E**; β-(1,3)-glucan (B^a,b,d^, light blue); non-reducing end β-(1,3)-glucan (NRb, dark blue); reducing end β-(1,3)-glucan (Rb, purple); mannan (M^a,b^, green), β-(1,6)-glucan (B^c^, bordeaux), α-(1,3)-glucan (NR^a^, rose); reducing end α-(1,3)-glucan (Ra^a,b^, pink).

For the identification of flexible cell wall components, we conducted scalar-based (INEPT-TOBSY) experiments (Fig. 4). The spectrum of the hydrated mycelium sample (Fig. 4A) displayed peaks from different sugar spin systems. Correlation patterns and our chemical shift analysis revealed mobile parts of β-(1,3)-glucan (B^a,b,d^) and α-glucan polysaccharides (NRa), which can be distinguished on the basis of their different C1 chemical shifts. Next to sugars tightly embedded in a polysaccharide (B ^a,b,d^), we found signals that possibly originate from the reducing (Rb, Ra^a-b^) and non-reducing ends (NRb, NRa) of these glucans. The reducing ends can be distinguished by a significant lower shift of their C1 when not connected. Notably, for the reducing ends Ra^a-b^ as well as the polysaccharide-monomers (B^a,b^) we did not obtain the C3 as it is connected to the polysaccharide and therefore more rigid than the rest of the spin system. Washing the cell envelope with PBS resulted in an increased number of signals compared to untreated, hydrated material (Fig. 4B). This observation indicates an increased accessibility of the cell envelope components to water, most likely due to the removal of surface-exposed cell envelope constituents. The components that appear after PBS washing are likely to be located closer to the rigid core of the cell envelope. Additional to new non-reducing ends of α-glucans (C1, C4 of NRa), more signals were obtained for the non-reducing ends of β-glucans (NRb, correlations to C1). Furthermore, the spectrum showed a new spin system, which, based on literature shifts and the HPLC results (vide infra), most likely corresponds to mannose (M^a^). However, as the C1 is missing this molecule may reflect a glucan in a different chemical environment (e.g. different hydration, conformation or cross-linkage). Interestingly, this preparation also revealed peaks for the C3 of the polysaccharide units (B^a,b^) while, at the same time, we observed intensity loss for peaks originating from reducing ends (R^b^, R^a^). This finding may suggest that less cross-connected polysaccharide chains start to dissolve. Indeed, the SDS treatment leads to full removal of these polysaccharides (Fig. 4C) as revealed by the disappearance of signals of reducing ends of glucans (R^a^, R^b^). Also, peaks that had been assigned to mannose disappeared. Instead, a different mannose (M^b^) species seems to become mobile. This mannose species is probably more cross-connected and in closer contact with the core of the cell envelope. New glucan species became mobile with chemical shifts pointing to reducing and non-reducing ends (NRa and Ra^b^), suggesting step-by-step loosening of glucan layers resulting from the increasingly destructive chemical treatment of our fungal material. Signals belonging to β-(1,3)-glucan polymers intensify upon SDS treatment confirming increased mobility of core-associated polymers. This suggests removal of polysaccharides that are not associated with the core of the cell envelope, so that the relative amount of β-(1,3)-glucan compared to other carbohydrates in this sample was higher than before.

In the spectrum of the alkali treated sample only peaks corresponding to the backbone β-(1,3)-(1,6)-glucan were found in line with the observation of flexible parts of the carbohydrate backbone (Fig. 4D). In addition to polysaccharide units, we could also detect remaining non-reducing ends of β-glucan (NRb) and a sugar lacking the resonances of C1 and C2 (B^c^). This could be due to missing dynamics of these atoms because they are integrated into the polysaccharide. Chemical shifts of the remaining mobile carbon atoms in this spin system suggest the presence of an (1,6)-linkage in these branched polymers or a role in cross-linking the polymers present (Dong et al., 2002). These linkages are also found in high abundance in the glycosyl linkage composition analysis (vide infra). Together, it is highly likely that the spectrum of the alkali-extracted sample (Fig. 4D) reflects molecular components of the cell envelope core-associated β-(1,3)-(1,6)-glucan.

In summary, we found that two different polymorphic forms of β-(1,3)-glucan (B^a,b^), β-(1,6)-glucan (B^c^) and non-reducing ends (NRb) of beta-linked glucan were stable towards all treatments and maintained their mobility, suggesting that they are mobile domains or surface species of the otherwise highly cross-connected, rather rigid core. Parts of alpha-linked non-reducing ends (NRa^a^) of α-glucan, one polymorphic form of alpha reducing ends (Ra^a^) of α-glucan and mannose M^a^ were stable to washing with PBS buffer, but were removed by SDS and alkali treatment, likely because they are less tightly associated or represent segments of supramolecular structures that undergo disruption due to the SDS treatment. A fraction of a β-(1,3)-(1,6) linked glucan (B^d^) became visible only after treatment with PBS, remained detectable after the SDS treatment and was washed out by the alkali treatment. Presumably the relative concentration of this species in the sample is only high enough for detection by NMR after PBS treatment. Another polymorphic form of reducing ends of α-glucan (Ra^b^) and another form of mannose (M^b^) were only detected in the SDS treated sample due to their increased relative abundance within the sample.

Lastly, we note that NMR signals in spectral regions characteristic for peptides or proteins only appeared in the spectrum of the untreated sample (see Fig. S2). Peak doubling for most of the amino acid signals would be consistent with either different polypeptides and/or polypeptides that adopt different structures. Treatment with PBS removed all signals, confirming previously found results in spectra recorded to study the rigid cell wall constituents.

### 3.2 Monosaccharide analysis via HPLC

To validate our ssNMR findings on the composition of the fungal cell wall we performed a monosaccharide analysis on a hydrolyzed sample. Mycelium was hydrolyzed using sulphuric acid and subsequent analysis with HPLC confirmed the presence of glucose (68%), mannose or galactose (9.1%) and fucose (1.3%) (Fig. S1). Mannose and galactose are not distinguishable in the HPLC spectra, however, ^13^C chemical shifts of the signals related to this molecule that were observed in our ssNMR spectra strongly suggest the presence of mannose and not galactose. Finally, N-acetylglucosamine could not be analyzed unambiguously due to signal overlapping with the solvent used in the HPLC measurements.

### 3.3 Glycosyl linkage analysis using GLC-MS

To gain more insight into the glycosidic linkages glycosyl linkage analysis was performed using a method developed by Anumula and Taylor (Anumula and Taylor, 1992) (Figs. S10-S31). Polysaccharides were methylated, hydrolyzed and subsequently reduced at C1 with NaBD4 enabling for the inerrant distinction of C1 and C6. Finally, the free hydroxy groups, that were involved in the polysaccharide linkages, are acetylated to produce partially methylated alditol acetates (PMAA). These PMAAs can be identified by their distinct fragmentation patterns in GLC-MS. The base used in this method was prepared according to Black et al., (Black et al., 2019). The analysis was carried out according to known procedures (Kamerling and Gerwig, 2007). Unfortunately, results obtained from the glycosyl linkage analysis do not reveal differences between α- or β-linkages under the chosen conditions. The linkage analysis of the four differently treated samples showed novel insights in differently linked hexoses and N-acetylhexoses (Table 2). This confirmed that not only the glucan core structure but also chitin is highly branched (SI Fig. S28-S.31). Besides (1,3)-, (1,4)-, (1,6)- and (1,3)-(1,6)-branched sugars we found as well small signals of sugars with branching degrees reaching up to 5 substitutions (Table 2). In the untreated sample (Fig. S10) (1,3)-linked hexoses gave rise to a major peak originating most likely from α- and β-(1,3)-linked glucan and also partially from the (1,3)-(1,6)-glucan core. Surprisingly, (1,4)-linked and terminal hexoses (Fig. S10, Fig. S16) were very abundant. In addition, we found (1,4)-linked N-acetylhexoses (e.g. chitin) albeit in lower amounts compared to the major species. Washing mycelium with PBS changed the composition of the material dramatically (Fig. S11), resulting in the most dominant peak in the chromatogram originating from (1,4)-linked N-acetylhexose. The (1,4)-linked hexoses were removed and the amount of (1,3)-linked glucans was strongly reduced. Yet, the (1,3)-(1,6)-glucans and hexoses with higher branching degrees were less affected by this treatment due to their enhanced stability. This relative composition changed again by treating the sample with hot SDS (Fig. S12). SDS extraction resulted in a relatively strong reduction of (1,4)-linked *N*-acetylglucans compared to the core glucan structures making them almost equally abundant over the entire sample. A possible explanation for this effect could be that SDS could disrupt the macromolecular structure of chitin to some extend making it more prone to solubilization (Pillai et al., 2009). Subsequent extraction of the sample with aqueous potassium hydroxide resulted in a strong loss of hexoses with high branching degrees like the (1,3–1,6)-glucans (Fig. S13) and a shift in the composition towards mainly chitin and branched chitin. A similar trend was observed in the ssNMR spectra. However, only the glycosyl linkage analysis revealed total loss of the (1,4)-linked hexoses in the first washing step of our material. Also, the hexoses and N-acetylhexoses with increased branching degrees were observed in our material for the first time (Fig. S13, Fig. S28-31) and chitins were not seen in the ssNMR spectra due to signal overlap.

**Table 2:** Glycosyl linkage analysis of hydrated, PBS-washed, SDS-extracted and alkali-extracted mycelium of *S. commune*. Abundances are given by peak area in percent calculated from the EI detector response. Preparational artefacts are grouped in ‘others’. They are observed in higher amounts for the alkali treated sample since we used less material for the analysis here.

Carbohydrate Linkage	Hydrated area %	PBS area %	SDS area %	KOH area %
hex*p*(1→	10.4	0.6	0.6	0.8
→3)hex*p*(1→	22.9	12.9/4.3	10.2/3.39	4.8
→4)hexp(1→	13.6	X	X	X
→6)hex*p*(1→	6.3/0.4	8.3/1.8	6.0	4.1
→3,4)hex*p*(1→	9.7	4.5	1.2/7.3	2.5
→2,3)hex*p*(1→	3.1	1.1	2.8	1.0
→2,4)hex*p*(1→	X	0.5	X	0.8
→4,6)hex*p*(1→	4.3	2.0	2.8	1.0
→3,6)hex*p*(1→	8.7	12.5/1.5/0.5	16.2/3.2	5.3
→2,3,4)hex*p*(1→	1.30	X	X	X
→3,4,6)hex*p*(1→	3.4	3.1	0.6/8.5	3.2
→2,3,6)hex*p*(1→	0.5	1.0	0.3/2.7	X
→2,4,6)hex*p*(1→	X	X	0.4	X
→2,3,4,6)hex*p*(1→	0.3	X	1.6	0.9
→4)hex*p*NAc(1→	3.4	20.2	14.3	22.6
→3)hex*p*NAc(1→	X	X	X	X
→3,4)hex*p*NAc(1→	0.3	2.6	2.0	2.8
→4,6)hex*p*NAc(1→	0.6	0.7/8.1	7.3	9.1
→3,4,6)hex*p*NAc(1→	X	0.8	0.9	0.9
other	10.9	13.1	8.0	60.0

## 4 Conclusions

Using a combination of ssNMR spectroscopy with HPLC and glycosyl linkage analysis we have shown that the main components of the cell wall of *S. commune* are different types of glucan, chitin, mannose and surprisingly also fucose. The rigid core seems to be built from different chitin forms ranging from highly branched chitin to single stranded β-(1,4)-chitin and α- and β-(1,3)-glucans including β-(1,3)-(1,6)-glucan. The presence of what we consider mannose and a yet unidentified sugar was only seen as long as mild treatments were used for washing. These two molecules seem to be part of a less tightly packed environment and could be either part of homopolysaccharides or ramifications on the other polysaccharide strands. However, we could not visualize a cross connection between different monosaccharides. Interestingly also fucose is strongly associated with the tightly packed inner core as it is still present after alkali treatment. The mobile fraction of the cell wall is composed of different surface species such as terminal hexoses in form of α and β-linked reducing and non-reducing ends of glucan. Here we also found different mannose species. Their presence in the mobile part of the cell wall points towards mannose as being part of an extended, mobile ramification or integrated into mannosylated proteins. Generally, the mobile fraction is more affected by the different washing steps than the rigid core, as it is less tightly packed and thereby more solvent accessible. Over the course of the different washing steps an increasing number molecules is removed leaving behind only the mobile part of the backbone β-(1,3)-(1,6)-glucan, which is the main component found in the *J*-based spectra of the alkali treated sample. Lipids and signals for amino acids or proteins were exclusively found in hydrated mycelium, indicating that they are not part of the cell wall but rather the inside of the cell.

**Fig. 5:**
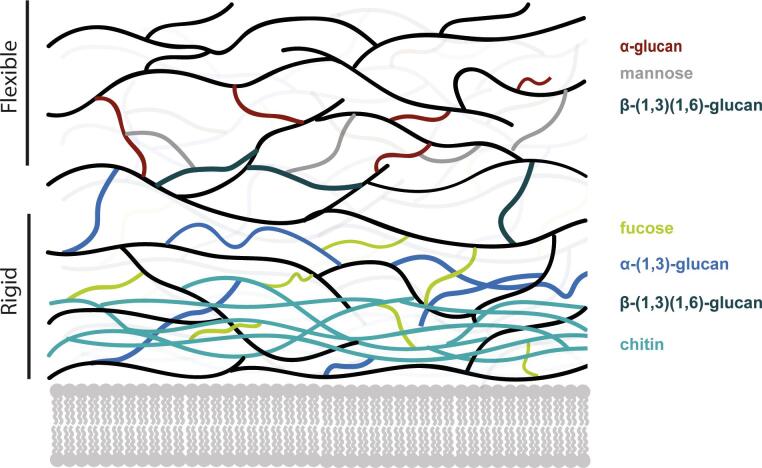
Model of the *S. commune* cell wall structure focusing on sugar composition. Adapted from Kang et al., 2018 (ref. (Kang et al., 2018)).

Based on our findings we were able to refine the current model for the composition and structural organization of the cell wall of *S. commune* (Fig. 5)*.* In part, it confirms what was already known or postulated for fungal cell walls of the model basidiomycete *S. commune* (Sietsma and Wessels, 1977, Sietsma and Wessels, 1981, van der Valk et al., 1977). However, compared to previous studies, our data suggest that the rigid part of the cell wall of *S. commune* not only contains chitin and β-(1,3)-(1,6)-glucan but also α-(1,3)-glucan and polymeric fucose*.* Finding both α-(1,3)-glucan as well as fucose to be present even after alkali treatment constitutes novel insight into the composition of the polysaccharide core structure of *S. commune.*

Alpha-(1–3)-glucan is found in the cell walls of both ascomycetes and basidiomycetes that represent the majority of fungal species. Notably, α-(1,3)-glucan was found for the first time in the core of a fungal cell wall in *Aspergillus fumigatus* (Kang et al., 2018). The fact that it is also present in the core of the cell wall of *S. commune* indicates that this polysaccharide plays an important structural role in the main phyla of the fungal kingdom. Thereby, it would be an interesting target for anti-fungal development. Indeed, an α-(1,3)-glucanase of *Trichoderma harzianum* was able to lyse cell walls of different plant pathogens (Ait-Lahsen et al., 2001). In contrast to α-(1,3)-glucan, fucose is not present in both main phyla of the fungal kingdom. Ascomycetes lack fucose (Crook and Johnston, 1962, Bartnicki-Garcia, 1968), while it is present in basidiomycetes and zygomycetes (Crook and Johnston, 1962, Bartnicki-Garcia, 1968, Bartnicki-Garcia and Lindberg, 1972). To our knowledge, this is the first time that fucose has been shown to play a role in the rigid alkali resistant part of the cell wall of a basidiomycete. It may be a target for anti-fungal development against basidiomycete pathogens or, when present in specific heteropolymers, it could be used for diagnosis.

No evidence was found for peptides being involved in polysaccharide cross-connections as previously described for *S. commune* (Sietsma and Wessels, 1979). The lack of protein signal was unexpected also as the fungal hydrophobin SC3 is known to form hot-SDS insoluble cell wall bound complexes (de Vries et al., 1993). Possibly, the relative abundance of SC3 compared to cell wall sugars is too small for the ssNMR methods used in our current study. Another explanation could be that the signals of the protein are not detected due to inhomogeneous broadening of the NMR lines caused by structural disorder or varying molecular interactions. More sophisticated methods such as DNP supported-solid state NMR (see, e.g. Ref. (Ni et al., 2013, Jantschke et al., 2015, Kang et al., 2018)) in conjunction with 3D ssNMR experiments including double-quantum filtered ^13^C spectroscopy (Heise et al., 2005) may be used in the future to increase ssNMR sensitivity and reduce spectral overlap. Morever, ssNMR methods that probe structure and macromolecular arrangements (Weingarth and Baldus, 2013) could be used further refine the molecular view of the *S. commune* cell wall. Together, we here showed the potential of ssNMR spectroscopy to provide atomic-level insight into the chemical composition and structure of complex biomolecules and, at the same time, its ability to differentiate between molecular domains exhibiting differential mobility. These spectroscopic properties allowed us to study the cell wall in its original structure and obtain molecular details, which are inaccessible for chemical treatment-based methods such as hydrolysis essays. A detailed understanding of the cell wall of *S. commune* and other fungi may provide novel targets for antifungals and diagnosis of plant, animal and human pathogens.

## Author contributions

FA prepared samples for NMR and HPLC. HLE & FA prepared samples for GC–MS analysis, HLE analysed GC–MS data. HLE, KH, MR recorded and analyzed the ssNMR data. All authors contributed to writing the manuscript and all authors have given approval to the final version of the manuscript.

## Declaration of Competing Interest

The authors declare that they have no known competing financial interests or personal relationships that could have appeared to influence the work reported in this paper.
