# Supplementary material for: Characterization of the cell wall of a mushroom forming fungus at atomic resolution using solid-state NMR spectroscopy
Source: Cell Surf. 2020 Oct 28;6:100046. doi: 10.1016/j.tcsw.2020.100046 (PMC7649524; doi:10.1016/j.tcsw.2020.100046)
Supplement: Supplementary data 1 [file mmc1.pdf]

Supporting data

Characterization of the cell wall of a mushroom forming fungus at atomic resolution using solid-state NMR

Supplementary Methods S1-4

Supplementary tables S1-S4

Supplementary Figures S1-S31

Supplementary References

Supplementary Methods

Methods S1: Sample preparation

Cultures were harvested from polycarbonate membranes and dried at ambient temperature. The mycelium was homogenized twice for 1 minute at 25 Hz in a stainless steel grinder jar containing a metal ball (20 mm diameter) using a Tissuelyser II (Qiagen, Hilden, Germany). The homogenized mycelium was hydrated using ultra-pure water to improve the NMR spectral resolution of this untreated fraction. Alternatively, the homogenized mycelium was washed twice with Phosphate Buffer Saline (PBS) (1.15 g $Na_2HPO_4.2H_2O$, 0.2 g KH_2PO_4, 0.2 g KCl, 8 g NaCl per liter, pH 7.4) followed by 3 washes with ultra-pure water. The material was centrifuged for 10 minutes at 6000 *g* (Eppendorf 5920R, Hamburg, Germany) between all washing steps. Part of this fraction was subjected to solid-state NMR analysis (see below), while the rest of the material was extracted 3 times in excess 1% SDS (Sigma-Aldrich, St Louis, MO, USA) for 10 min at 100°C, followed by 3 washes with ultra-pure water. Again, the material was centrifuged for 10 minutes at 6000 *g* between the washing steps. The resulting SDS extracted cell wall fraction was subsequently incubated in 1 M KOH (Sigma-Aldrich, St Louis, MO, USA) for 20 min at 60°C, after which the pH was set to 5 with acetic acid. The alkali insoluble cell wall fraction was pelleted 15 min at 6000 x *g,* washed 2 times with ultra-pure water for 1 min at room temperature by suspending and vortexing. This procedure was followed by a 12-h-washing step with ultra-pure water at 4°C, after which the alkali insoluble cell wall fraction was collected via centrifugation for 15 min at 6000 x *g*.

Methods S2: High Performance Liquid Chromatography

Sugar analysis was performed after total hydrolysis of the fungal cell walls with sulphuric acid. To this end, 0.1 g untreated cell wall fraction (see above) was taken up in 2 mL ultra-pure water at 4°C and centrifuged for 15 min at 4000 *g*. The pellet was suspended in 2% SDS, 40 mM ß-mercaptoethanol, 50 mM Tris-HCl, and 5 mM EDTA, pH 7.4. After incubating for 10 min at room temperature and centrifugation for 15 min at 4000 *g*, the pellet was resuspended in 1 mL ultra-pure water. After freeze drying, 15 mg of the material was suspended in 225 µL 72% (w/w) sulfuric acid (Sigma-Aldrich, St Louis, MO, USA). After 3 h incubation at room temperature, the sulphuric acid was diluted to 1 M by adding 2.81 mL ultra-pure water. This suspension was incubated for 4 h at 100 °C in a water bath. After the suspension was cooled to room temperature, pH was set at 6–8 with 40 g L^{-1} $Ba(OH)_2.8H_2O$ (Sigma-Aldrich, St Louis, MO, USA) and stored for 12 h at 4°C to allow precipitation of $BaSO_4$. After centrifugation for 15 min at 4000 *g*, metabolite concentrations were determined in the supernatant by HPLC using elution at 60°C with 0.6 mL min^{-1} 5 mM H_2SO_4 and an Aminex HPX-87H column (Biorad) connected to a Waters Alliance e2695 HPLC. Peaks were detected by a refractive-index detector (Waters 2414) and a dual-wavelength absorbance detector (Waters 2489) at 210 nm and 270 nm. Raw data were processed into PeakArea's and concentrations were determined with Empower2 software from Waters.

Methods S3: Glycosyl linkage analysis

Samples (3 mg) were mixed with 400 µL of DMSO and stirred for two days. Next, 400 µL of NaOH in DMSO was added to the samples and the resulting solutions were stirred for 2h. Another 400 µL of base was added and the solutions were stirred for ten more minutes. Then 100 µL of iodomethane was added to each sample and the reaction solutions were stirred for 40 minutes. The treatment with base and iodomethane was repeated once and then 2 ml of water was added, causing the samples to turn cloudy. Then a nitrogen flow was bubbled through the solutions to remove iodomethane. Once the solutions became clear the flow was removed. Subsequently, 2 mL of dichloromethane (DCM) was added, the test tubes were vortexed for 45 seconds and then centrifuged at 4000 x *g* for 5 minutes. The top layer was taken off then 2 mL of water were added to the tubes. After centrifugation (4000 *g*, 5 min) the top layer was removed, and the washing step was repeated. The residue was transferred to a different tube and dried. Cleavage of the permethylated polysaccharides was achieved by adding 400 µL of 4M HCl to each tube followed by incubation at 100 °C for 4h. The samples were dried and 2 mL of water was added and evaporated to remove residual HCl. The washing step was repeated two times. For the reduction, 10 mg of sodium borodeuteride was added to 1 mL of 1M ammonium hydroxide. Next, 400 µL of the resulting solution was added to the samples and the solutions was let at room temperature overnight. Afterwards 5 drops of glacial acetic acid and then 5 drops of methanol was added to each of the reaction solutions and then the reaction solutions were dried. Subsequently 400 µL of a 9:1 solution of methanol and acetic acid were added and dried. This step was repeated once. In the next step, 200 µL of methanol was added and dried. The addition and evaporation of methanol was repeated two times. For *O*-acteylation 250 µl acetic anhydride and 250 µL pyridine were added to each sample. The reaction solutions were incubated at 100 °C for one hour. Once they had cooled 2 mL of isopropanol was added and evaporated. Then 2 mL of each water and DCM were added and the tubes were vortexed for 30 seconds in order to extract the water layer. Subsequently the top layer was removed, and the DCM phase was washed with water twice. The bottom layer was transferred to a clean tube and dried. The residue was taken up in 400 µL DCM and analyzed by GLC-EI-MS (GCMS-QP2010 Plus, Shimadzu Kratos Inc., Manchester, UK) using an EC-1 column (30 m x 0.25 mm, Alltech/Grace, Deerfield, IL, USA) with a temperature gradient (140-250 °C at 8 °C/min).*

---

* of the alkali treated sample only 0.9 mg was weighed in

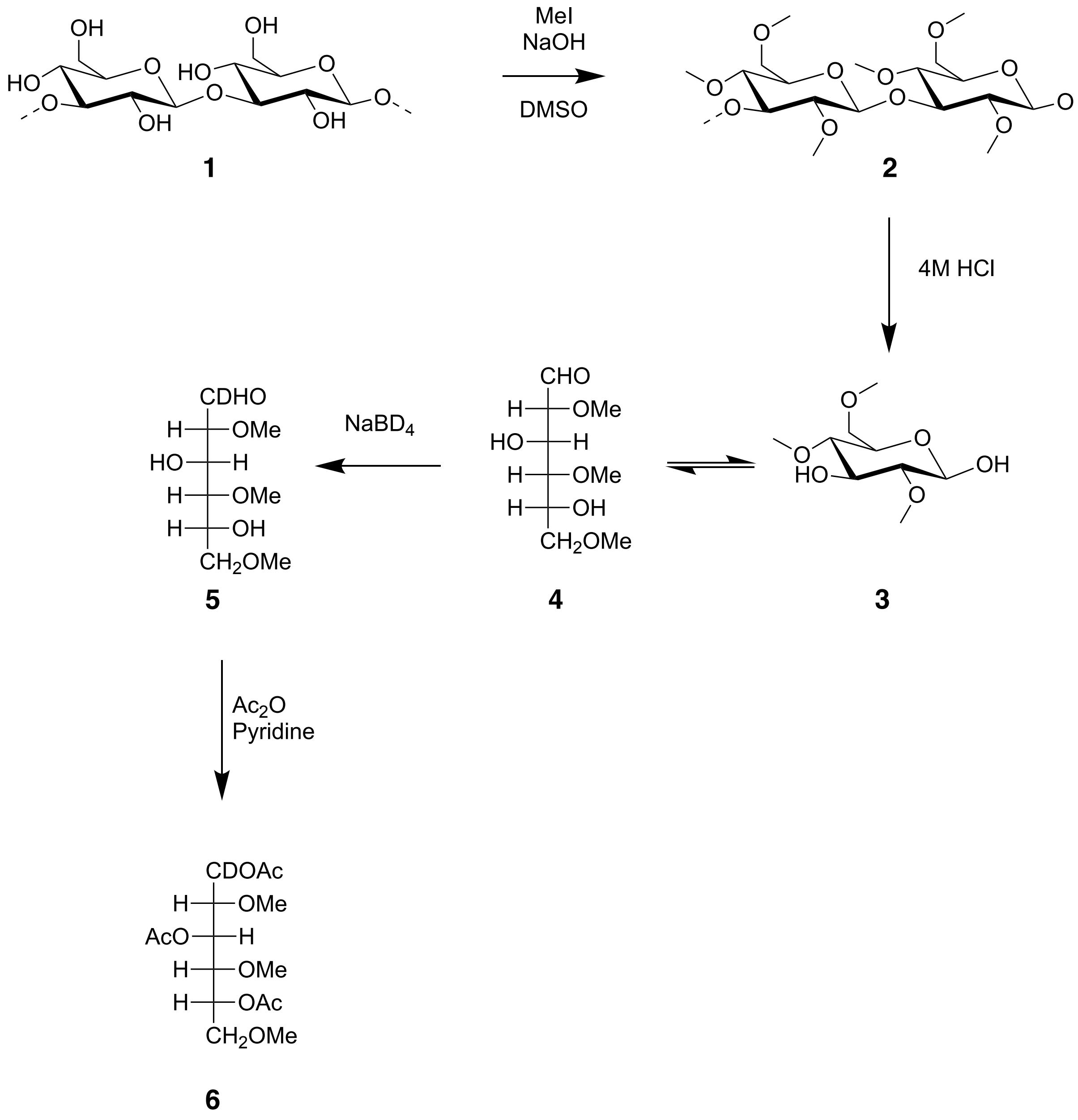

Scheme S1 Reaction Scheme for the formation of permethylated alditol acetates (PMAA). Polysaccharides are permethylated (**2**), hydrolyzed (**3**), reduced (**5**) and finally acetylated (**6**).

Methods S4: Solid-State NMR

Mycelium was transferred into standard 3.2 mm MAS rotors by low-speed centrifugation, packed with top and bottom spacers and subsequently analyzed by solid-state NMR (ssNMR) spectroscopy. Magic Angle Spinning (MAS) solid-state NMR (ssNMR) experiments were performed on a Bruker 700 MHz standard bore magnet equipped with an AVANCE-III console using a 3.2 mm MAS Efree HCN triple channel probe or on a 800 MHz wide bore magnet with an AVANCE-III console using a 3.2 mm HNCD MAS probe. Mycelium was filled into standard 3.2 mm MAS rotors. Spectra were recorded at a set temperature of 260 K and at MAS frequencies of either 12 or 13 kHz leading to sample temperatures above 0 °C due to frictional heating. ^{1}H, ^{13}C and ^{15}N chemical shifts were referenced externally to histidine and adamantane. ^{1}H and ^{13}C pulses were applied with 70–90 kHz and 45–50 kHz, respectively. For 1D and 2D ^{13}C dipolar-based NMR experiments, high-power proton decoupling using SPINAL64 (Fung, Khitrin, & Ermolaev, 2000) with an rf amplitude of 70–90 kHz was applied during evolution and detection periods. For all experiments employing through-bond magnetization transfer steps (J-based experiments), a low-power ^{1}H decoupling using WALTZ (Shaka, Keeler, & Freeman, 1983) with an rf amplitude of 10 kHz was applied during the detection period. 1D spectra were recorded using a 2 s recycling delay and acquisition times of 8–9 ms and 8–17 ms for dipolar-based and *J*-based NMR experiments, respectively. Cross-polarization (CP) conditions were optimized using 70–100% ramped ^{1}H pulses, corresponding ^{13}C pulses and contact times between 400 and 1000 μs (Table S1). 1D direct excitation (DE) and CP spectra were processed with line-broadening of 10–30 Hz and 50–100 Hz for the INEPT (Insensitive Nuclei Enhanced by Polarization Transfer (Morris & Freeman, 1979) spectra. The dipolar based 2D ^{13}C–^{13}C Spin diffusion experiments were recorded using a 2 s recycling delay and 10 ms and 4–7 ms acquisition times in the direct dimension (F2) and the indirect dimension (F1), respectively. ^{13}C-^{13}C mixing was propagated through proton driven spin diffusion using Phase-Alternated-Recoupling-Irradiation-Schemes (PARIS) (Weingarth, Bodenhausen, & Tekely, 2010) for 120 ms. The resulting spectra were processed with a 0.25 π shifted sine squared window function in both dimensions. *J*-based INEPT-TOBSY (Total-Through-Bond-Correlation-Spectroscopy) spectra (Andronesi et al., 2005; Baldus & Meier, 1996) were recorded using 6.1 ms mixing time at 145 Hz, 1.5 ms recycle delay and acquisition times of 13–17 ms (F2) and 6-9 ms (F1), respectively. A 0.33 π shifted sine squared window function in both dimensions was used for processing these spectra. Additional homonuclear double-quantum (DQ) – single quantum (SQ) correlation experiments involved SPC5 (Hohwy et al., 1999) recoupling. Heteronuclear correlation experiments involved SPECIFIC-CP (Baldus et al., 1998) to transfer polarization from ^{15}N to ^{13}C nuclei. All Spectra were processed

and analyzed using BRUKER Topspin 3.5 and analysis was done with SPARKY (Goddard and Kneller, SPARKY 3, University of California, San Francisco). Chemical shifts reported in the literature (Table S3) were used to assist resonance assignment of the ssNMR spectra.

Supplementary Tables

Table S1. Acquisition parameters for the carbon detected 1D and 2D experiments.

Sample	1H pulse (kHz)	13C pulse (kHz)	Dec. SPINAL64 (kHz)	CP contact time (μs)	13C CP-field (kHz)	1H CP-field (kHz)	1D DE ns	1D CP ns	1D INEPT ns	2D PARIS ns	2D INEPT ns
**Hyd.**	71	46	71	400	42	63	512	512	512	128	16
**PBS**	83	50	83	600	47	83	64	64	64	48	128
**SDS**	87	50	87	1000	44	70	128	128	128	96	64
**Alk.**	71	50	71	800	45	87	32	256	128	176	16

Table S2 Acquisition parameters for the nitrogen detected and nitrogen filtered 1D and 2D experiments.

Sample	Type of spectrum	1H pulse (kHz)	15N pulse (kHz)	Dec. SPINAL64 (kHz)	CP contact time (μs)	15N CP-field (kHz)	1H CP-field (kHz)	13C CP-field (kHz)	ns
Hyd.	1D HN-CP	71	46	71	400	42	63	-	512
Hyd.	2D NCA	71	46	71	HN: 1050<br>NC: 3400	HN: 34<br>NC: 19	54	8	512
Hyd	2D NCO	71	46	71	HN: 1050<br>NC: 3000	HN:34<br>NC: 19	56	8	3712

Table S3. Carbon Chemical shifts in ppm of different sugars according to literature.

	abbreviation	C1	C2	C3	C4	C5	C6	C7	C8	References
β-1,3-glucan[1]		103.3	73.9	86.3	68.7	76.2	61.1			Chakraborty et al 2006
β-1,3-glucan[2]	B[a,b,d]	102.7	72.7	86.0	68.3	76.2	60.9			Alquini et al 2004
β-1,3-glucan[3]		105.3	75.9	87.0	70.8	78.3	63.4			Fontaine 2000
Reducing end β-1,3-glucan	Rb	98.4	76.5	87.4	70.9	78.3	63.5			Fontaine 2000
Non-reducing end β-1,3-glucan[1]		104.4	75.2	77.8	71.8	78.1	62.9			Dong et al 2002
Non-reducing end β-1,3-glucan[2]	NRb	103.1	73.8	76.5	70.3	76.3	61.0			Chang and Lu 2004
Non-reducing end β-1,3-glucan[3]		105.5	76.2	78.3	72.3	78.7	63.4			Fontaine 2000
α-1,3-glucan[1]	A[a]	101.0	71.4	83.2	70.3	73.5	61.3			Bock et al 1983
α-1,3-glucan[2]		97.0	70.3	80.1	69.8	73.4	61.8			Mondal et al
Reducing end α-glucan	Ra[a,b]	94.7	73.8	85.2	70.8	73.9	63.3			Fontaine 2000
Non-reducing end α-glucan[1]		99.7	71.8	72.8	70.0	71.3	60.8			Smiderle et al.
Non-reducing end α-glucan[2]	NRa	101.6	75.7	76.1	74.4	75.6	63.4			Pang et al.
Non-reducing end α-glucan[3]		98.7	72.1	74.0	69.7	72.7	-			Yalin et al.
α-1,6-glucan[1]		100.2	72.0	72.7	73.9	75.9	68.1			Luo et al 2008
α-1,6-glucanP[2]		101.1	72.1	74.0	70.6	71.3	67.6			McIntyre and Vogel 1993
α-1,4-glucan[1]		99.8	71.5	75.3	78.7	71.2	60.1			Gonzaga et al 2005
α-1,4-glucan[2]		100.7	72.1	74.2	77.7	72.1	61.5			McIntyre and Vogel 1993
Mannan[1]		102.0	76.3	72.5	81.4	70.3	62.4			Marchessault et al 1990
Mannan[2]		102.4	75	72.5	83	70.9	62.9			Marchessault et al 1990
Mannan[3]	M[a,b,c]	101.7	69.9	72.1	81	75.9	61.9			Petkowicz et al 2001
Mannan[4]		101.6	78.9	70.9	67.9	74.1	61.9			Gómez-Miranda et al. 2003
Mannan[5]		99.3	79.4	71.4	67.7	73.7	66.5			Gómez-Miranda et al. 2003
β-1,3-galactan		105.7	72.4	82.2	70.2	76.6	62.5			Bilan et al 2010
β-1,6-galactan		105	72.3	74.2	70.2	75.2	71.5			Bilan et al 2010
Chitin[1]		104.2	55.2	74.8	84.1	74.8	60.9	173.1	22.5	Jang et al 2004
Chitin[2]	Ch[a,b,c]	104.0	54.6	73	82.8	75.4	60.5	172.6	22.5	Jang et al 2004
Chitin[3]		104.3	55.3	73.5	83.2	75.9	61.1	175.8-173.5	22.9	Kameda et al 2004
α-1,3-fucan	F	97.2	68.1	76.3	70	68.1	16.8			Bilan et al 2010
β-1,6-glucan	B[c]	105.1	75.2	77.8	71.8	77.1	71.0			Dong et al. 2002

Table S4. Chemical shifts (ppm) of peaks shown in Figure S2.

	a	b	c	d	e	f	g	h	i	j	k	l	m	n	o	p	q
Figure 2A	16,7	17,9	23,1	25,3	27,4	29,8	32,3	34,5	36,2	74,1	75,8	87,6	94,8	98,6	174,7	176,6	178,4
Figure 2B	16,8	25,4	27,5	Na	29,8	32,3	34,5	36,2	65,3	74,2	77,0	94,9	98,6	Na	130,5	132,2	
Figure 2C	16,9	23,1	24,9	32,6	35,2	42,0	57,6	66,2	76,0	78,3	80,3	88,52	105,9	176,7			

Supplementary Figures

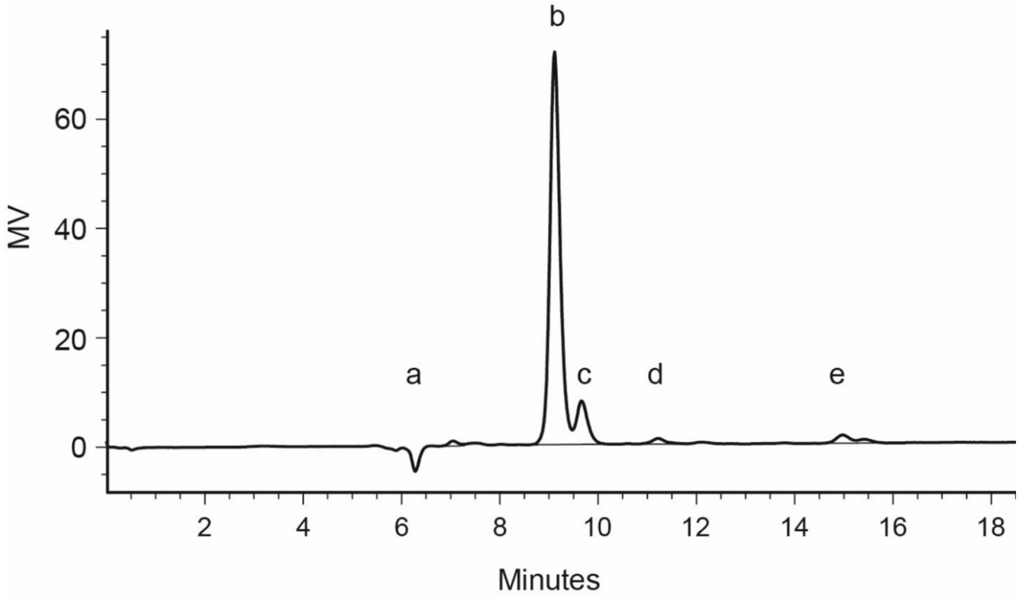

Figure S1. HPLC Chromatogram obtained for *S. commune* mycelium. a) 9.299 min, negative peak representing an artefact of the eluent. This is also the retention time of *N*-Acetylglucoseamine that is now invisible. b) 9.133 minutes, glucose; c) 9.681 minutes, mannose and/or galactose; d) 11.241 minutes, fucose; e) 14.994 min, acetic acid possibly arising from sulfuric acid hydrolysis of acetyl group from chitin.

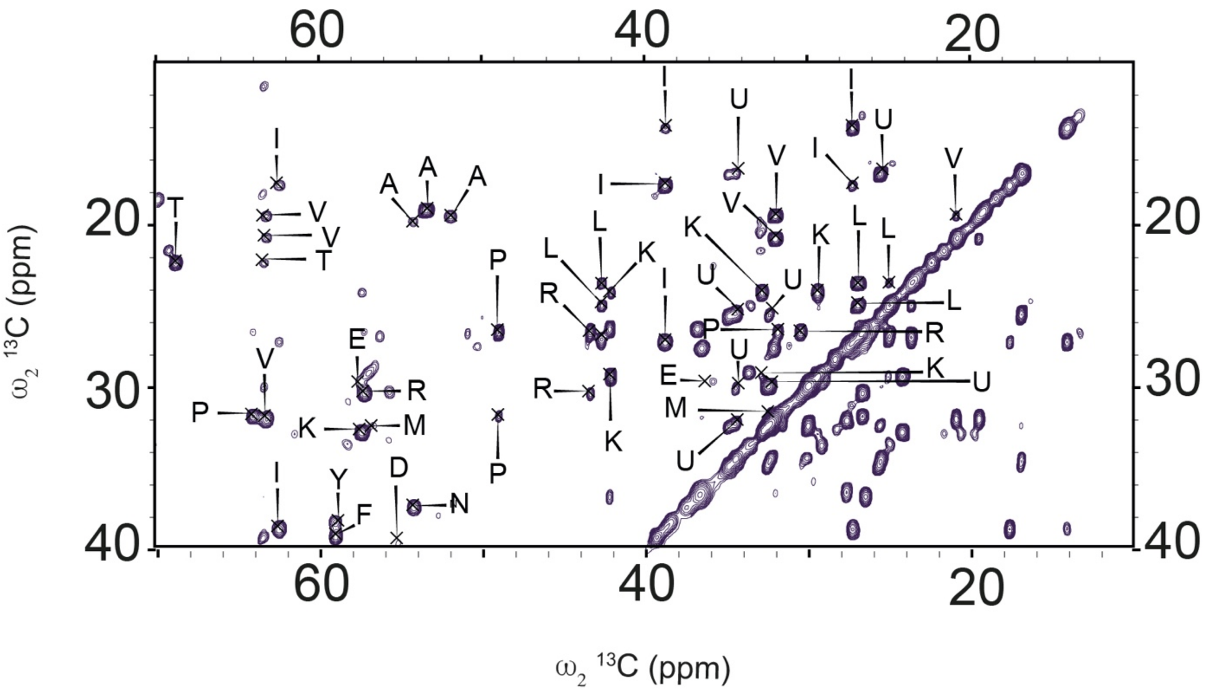

Figure S2 Spectral cutout of 2D (^{13}C,^{13}C) INEPT-TOBSY of hydrated mycelieum showing the amino acid region. Resonance assignments are indicated in the spectrum using the one-letter code for amino acids.

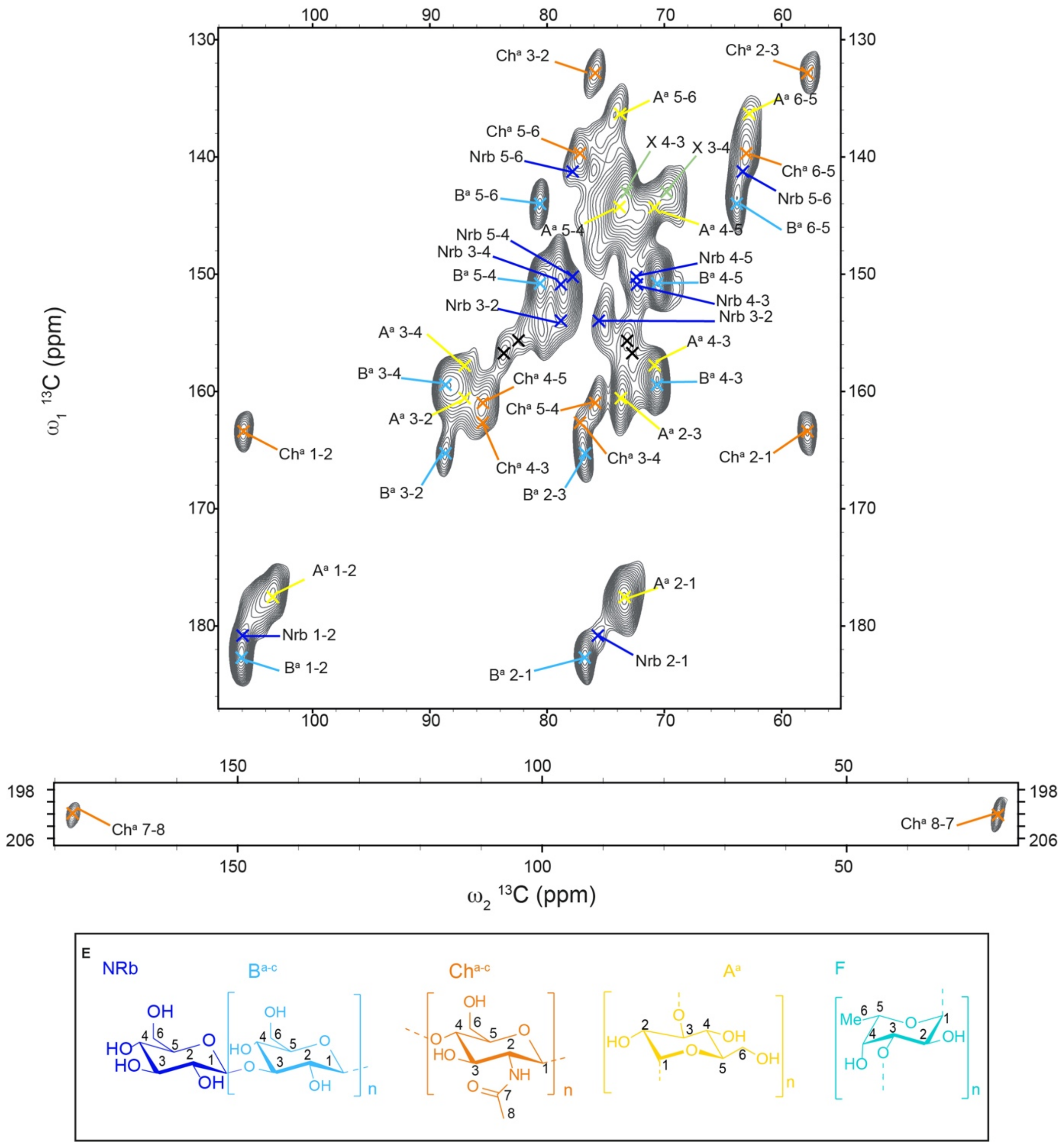

Figure S3. Two dimensional ^{13}C - ^{13}C double-quantum single-quantum (DQSQ) spectrum of the hydrated (U-^{13}C,^{15}N)-labelled mycelium of *S. commune*. Peak annotations correspond to the molecules shown in panel **E**. The annotation X corresponds to an unidentified molecule.

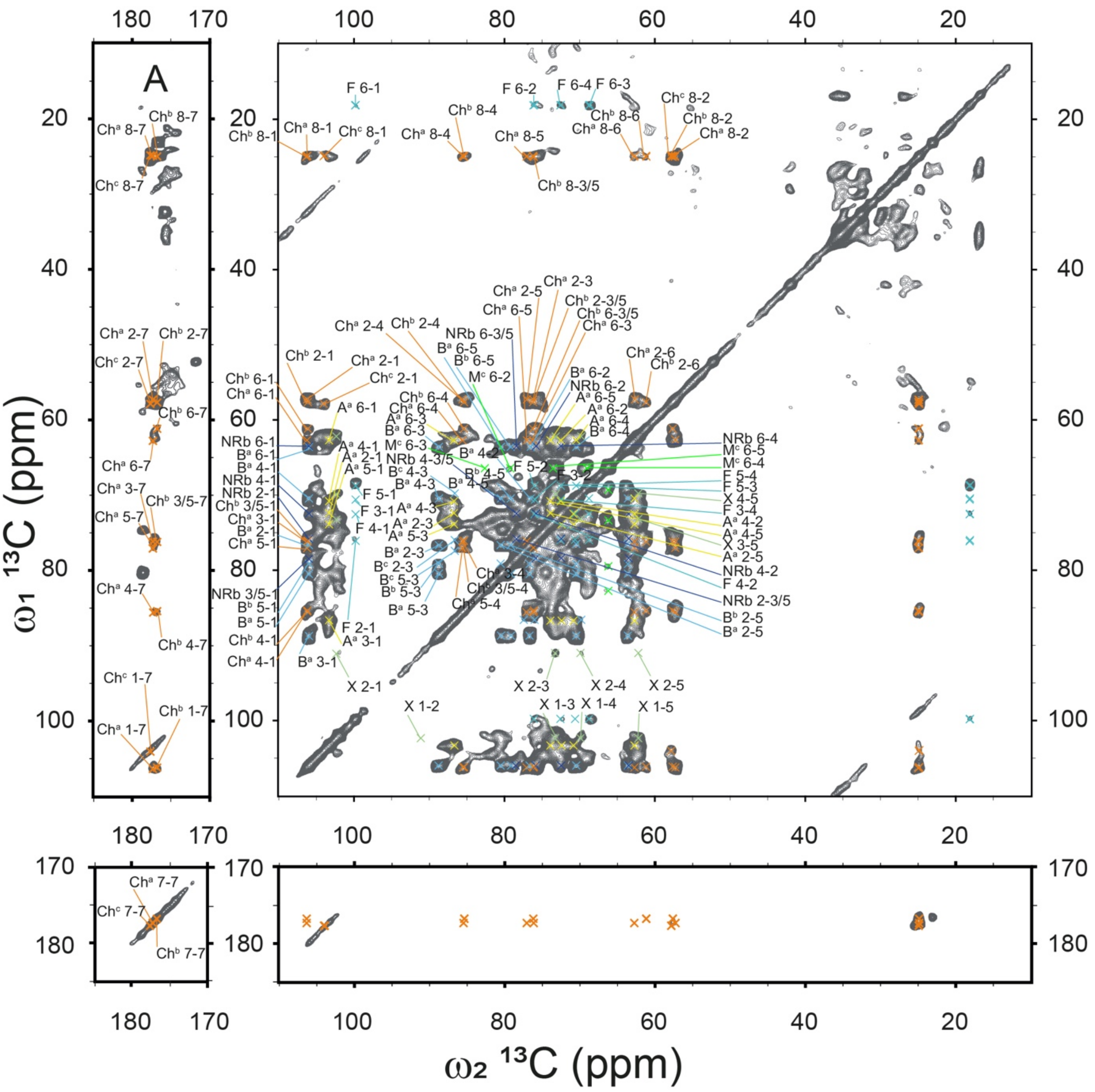

Figure S4. Two-dimensional solid-state ^{13}C-^{13}C spin-diffusion spectrum of non-treated *Schizophyllum commune* mycelium recorded with CP magnetization and PARIS recoupling (mixing time: 120 ms). Identified spin systems are marked in colors as follows: β-1,3-glucan (B^{a-c}, light blue); non-reducing end β-1,3-glucan (NRb, dark blue); chitin (Ch^{a-c}, orange); fucan (F, turquoise); α-(1,3)-glucan (A^a, yellow). ^{13}C chemical shifts are reported in Table S1.

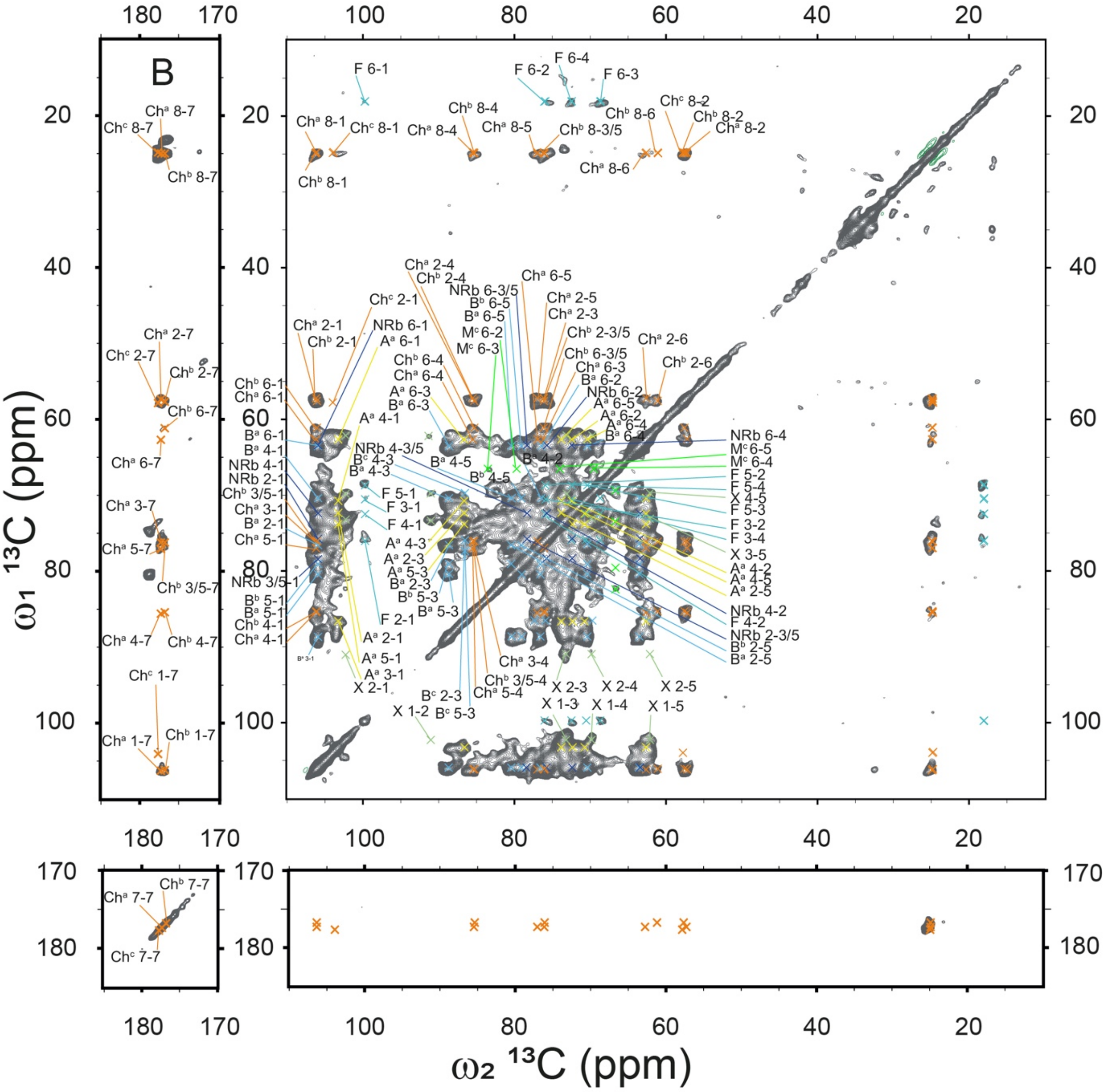

Figure S5. Two-dimensional solid-state ^{13}C-^{13}C spin-diffusion spectrum of PBS-treated *S. commune* mycelium recorded with CP magnetization and PARIS recoupling (mixing time: 120 ms). Identified spin systems are marked in colors as follows: β-1,3-glucan (B^{a-c}, light blue); non-reducing end β-1,3-glucan (NRb, dark blue); chitin (Ch^{a-c}, orange); fucan (F, turquoise); α-(1,3)-glucan (A^{a}, yellow). ^{13}C chemical shifts are reported in Table S3.

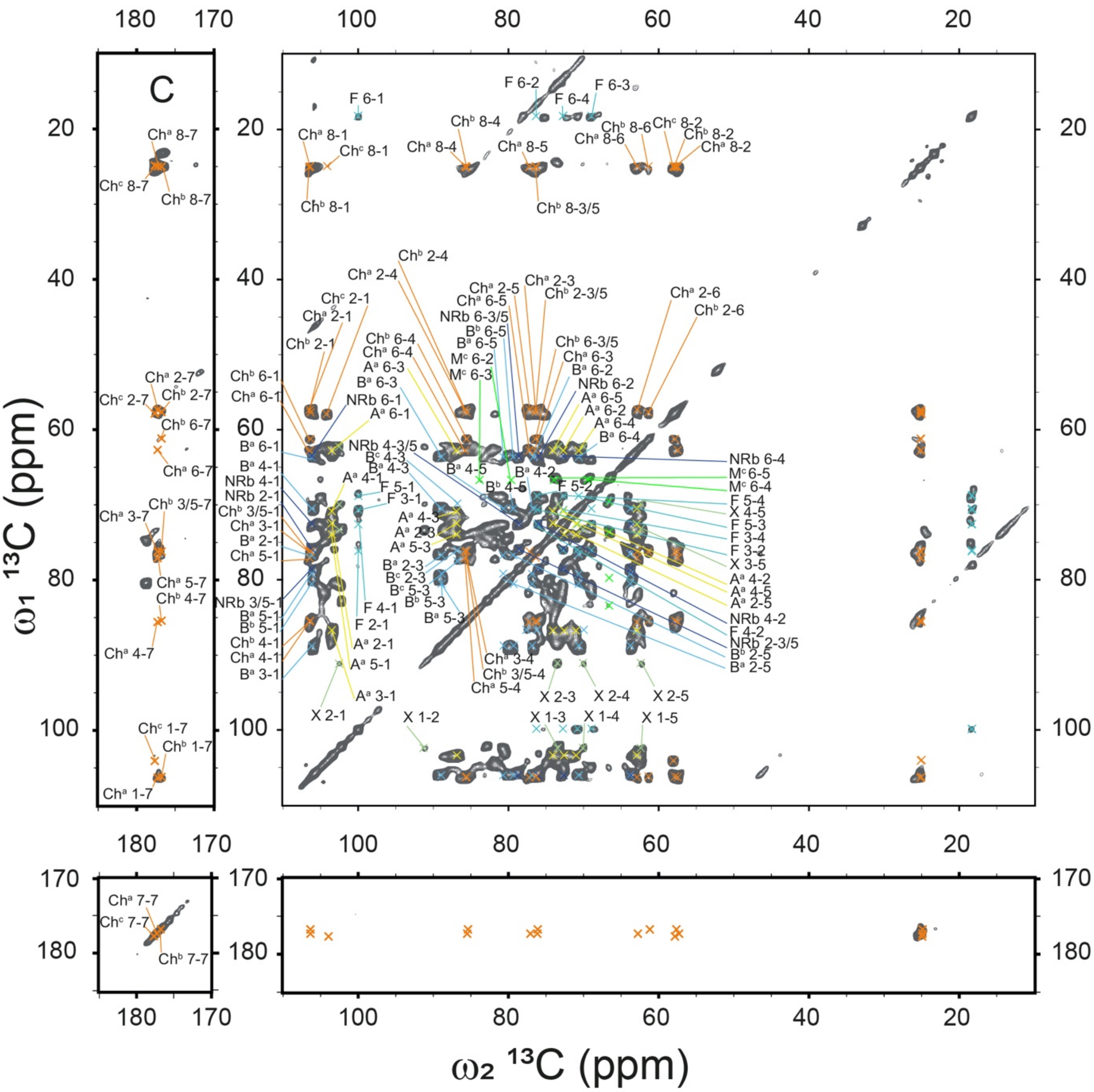

Figure S6. Two-dimensional solid-state ^{13}C-^{13}C spin-diffusion spectrum of SDS-treated *S. commune* mycelium recorded with CP magnetization and PARIS recoupling (mixing time: 120 ms). Identified spin systems are marked in colors as follows: β-1,3-glucan (B^{a-c}, light blue); non-reducing end β-1,3-glucan (NRb, dark blue); chitin (Ch^{a-c}, orange); fucan (F, turquoise); α-(1,3)-glucan (A^a, yellow). ^{13}C chemical shifts are reported in Table S1.

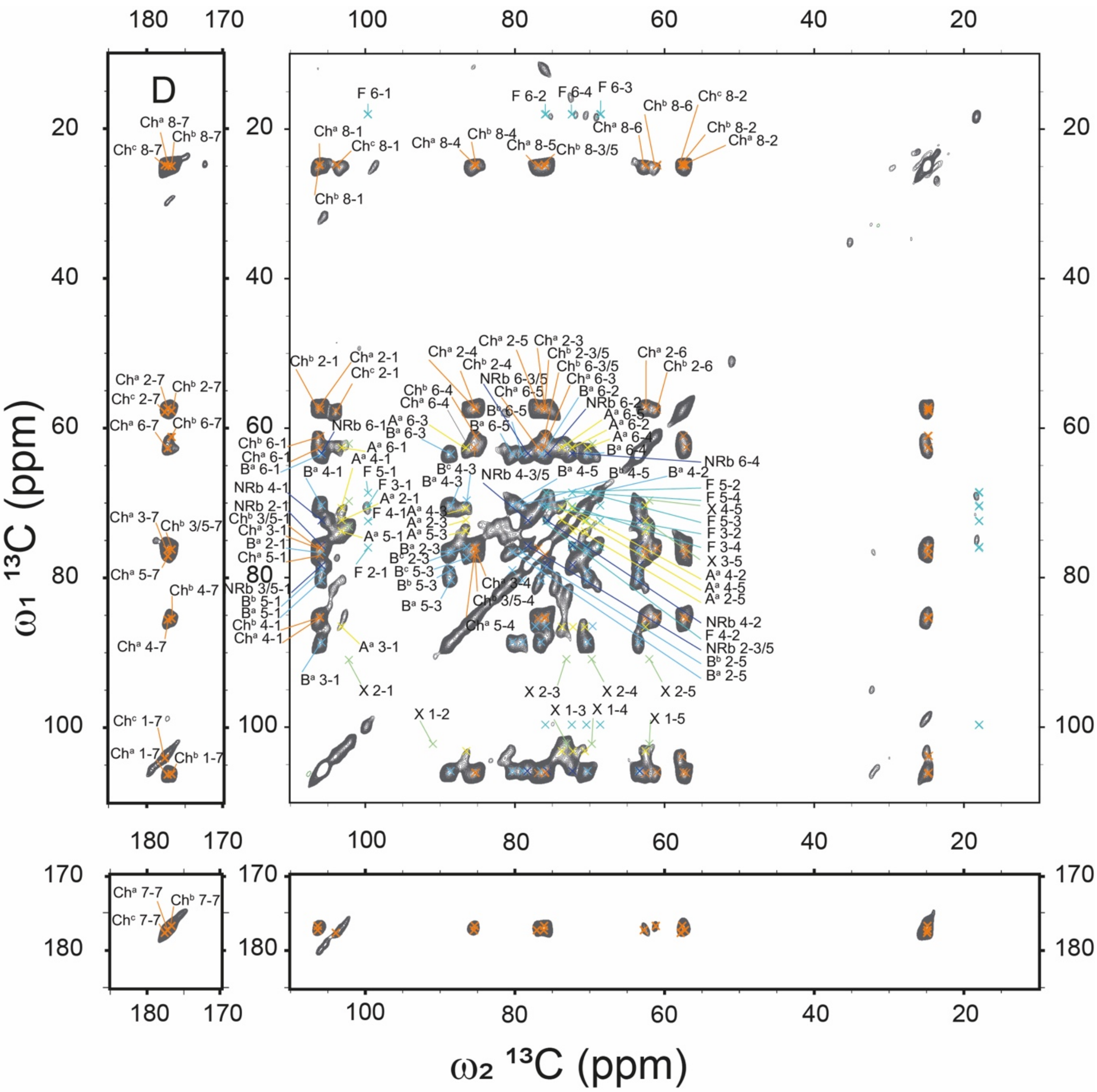

Figure S7. Two-dimensional solid-state ^{13}C-^{13}C spin-diffusion spectrum of alkali-treated *S. commune* mycelium recorded with CP magnetization and PARIS recoupling (mixing time: 120 ms). Identified spin systems are marked in colors as follows: β-1,3-glucan ($B^{a\text{-}c}$, light blue); non-reducing end β-1,3-glucan (NRb, dark blue); chitin ($Ch^{a\text{-}c}$, orange); fucan (F, turquoise); α-(1,3)-glucan (A^a, yellow). ^{13}C chemical shifts are reported in Table S1.

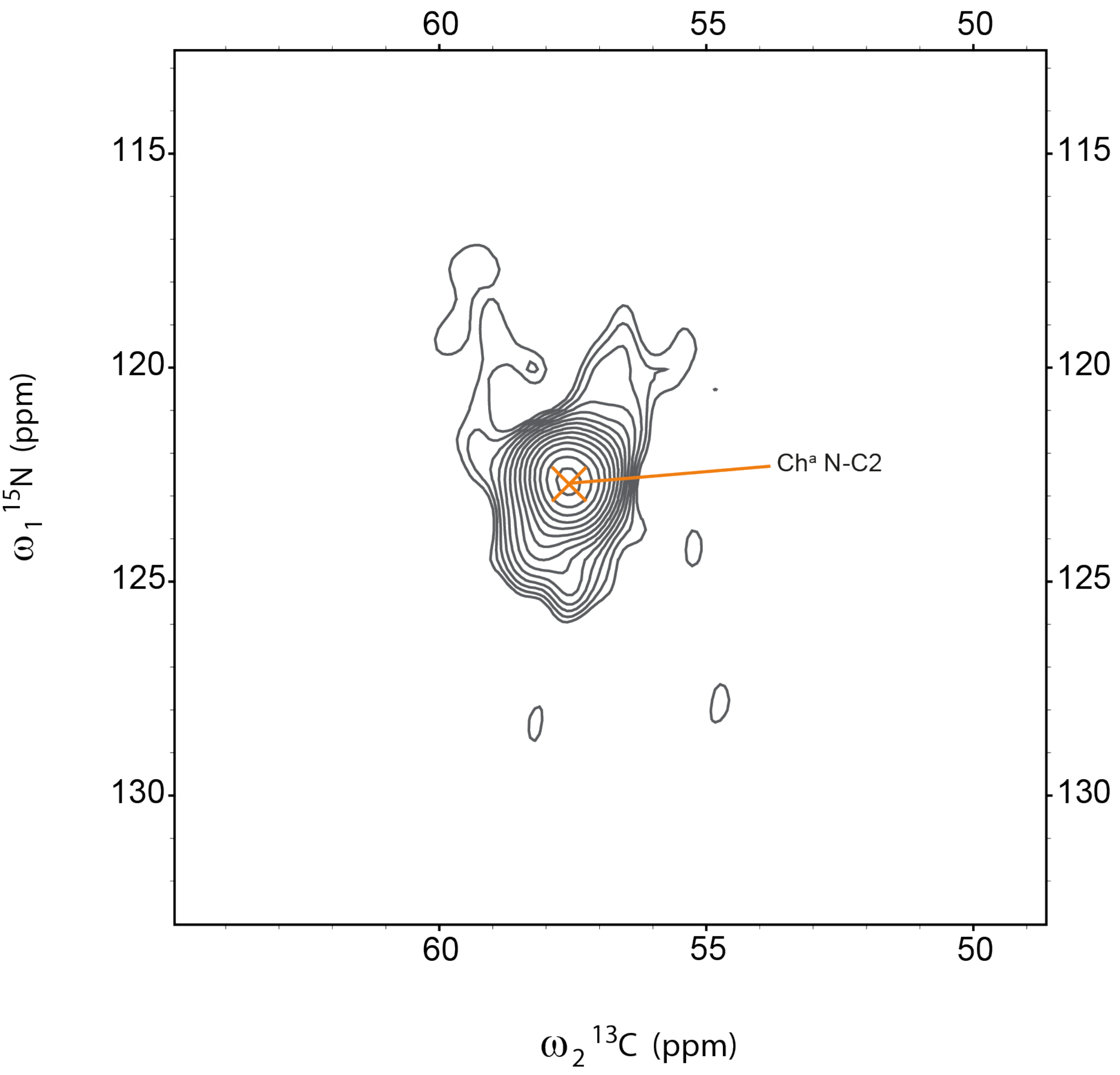

Figure S8. ^{13}C-^{15}N correlation spectrum of the non-treated mycelium of *S. commune*. After a CP step from ^{1}H to ^{15}N, SPECIFIC-CP transfer was established to the Cα carbons. The peak shown corresponds to the correlation of chitin C2 with the attached amide HN. Amino acid signals were not detected.

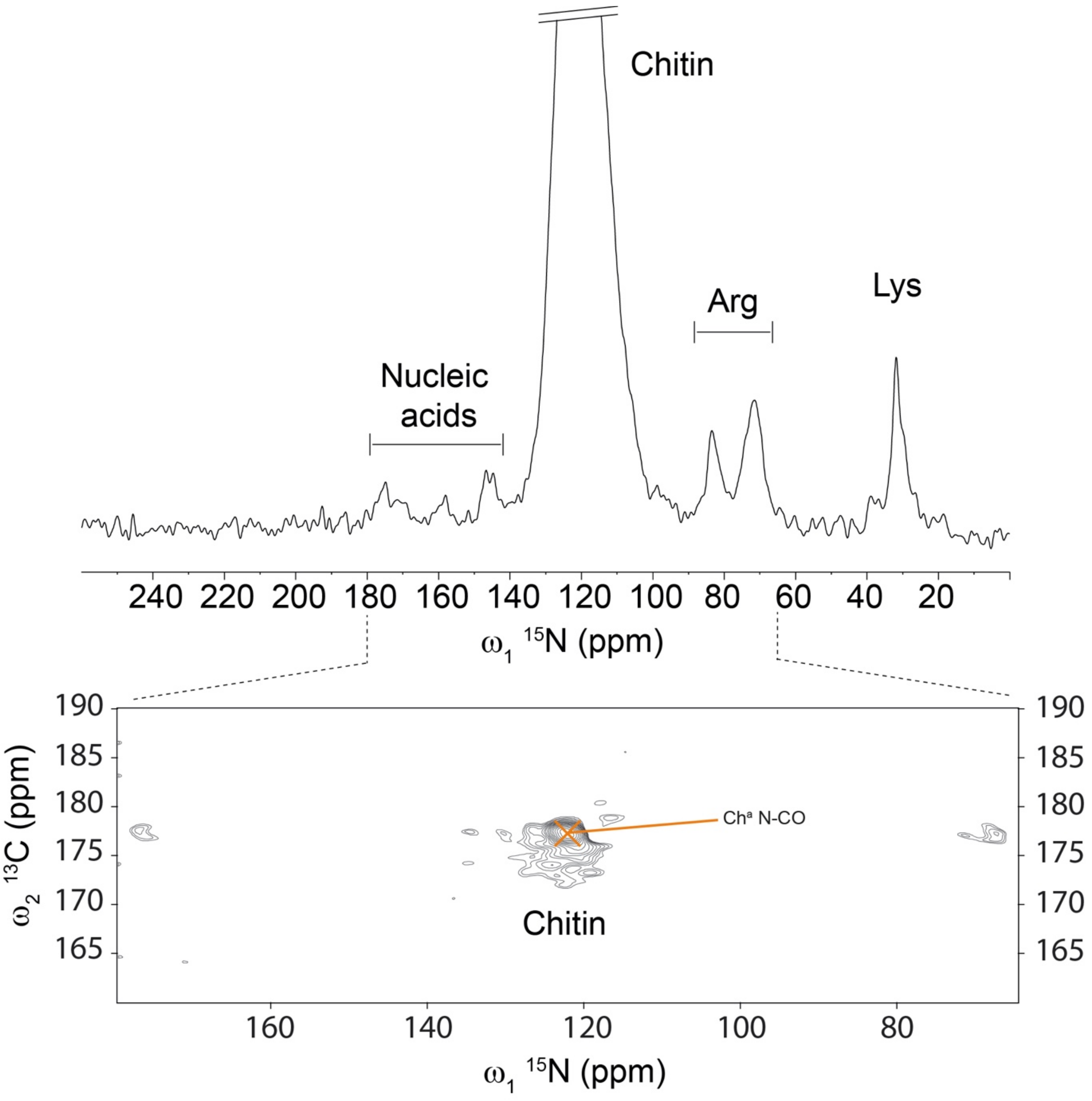

Figure S9. ^{13}C-^{15}N correlation spectrum of the non-treated mycelium of *S. commune*. After a CP step from ^{1}H to ^{15}N, SPECIFIC-CP transfer was established to the CO carbons. The peak shown corresponds to the correlation of chitin CO with the attached amide HN. Amino acid signals were not detected in the 2D spectrum. The 1D ^{15}N-CP-spectrum was recorded with 16k scans and shows signals belonging to chitin, side chains of arginine and lysine and nucleic acids.

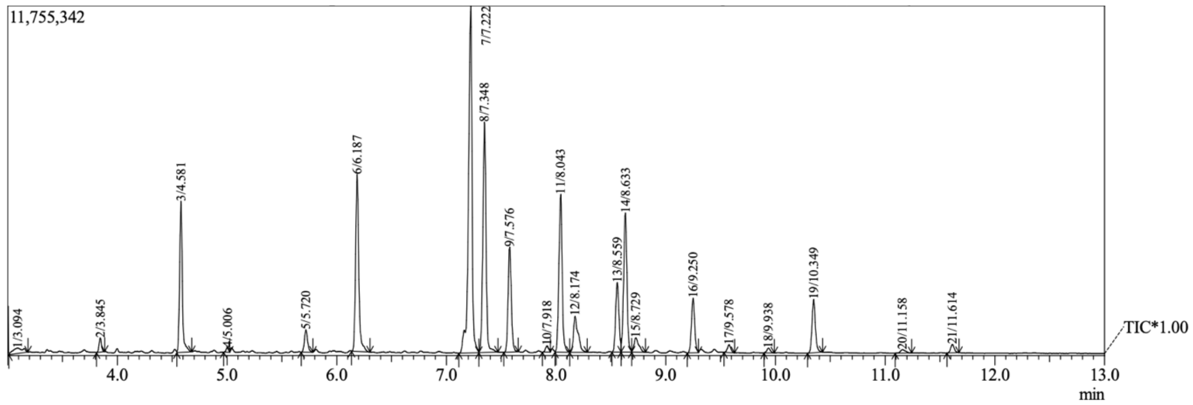

Figure S10. GLC-Chromatogram of untreated mycelium of *S. commune*. Peak numbers are indicated together with elution times.

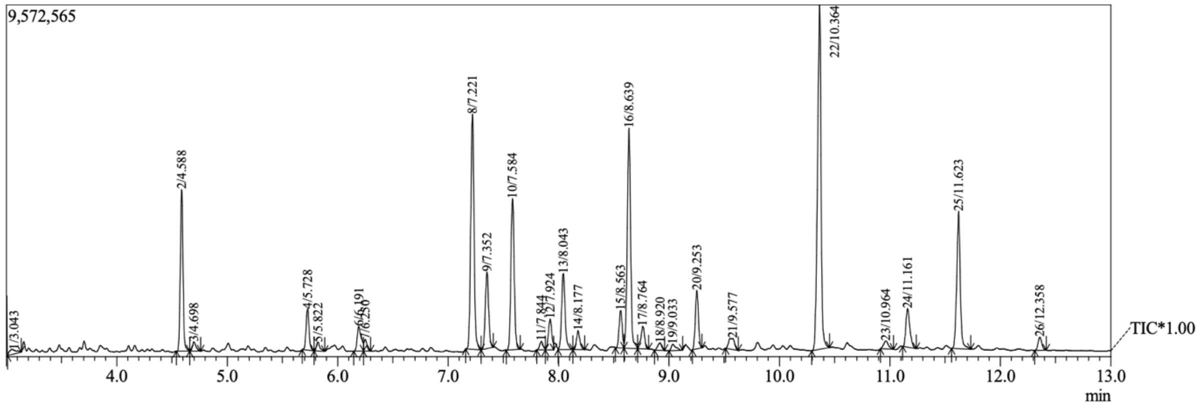

Figure S11. GLC-Chromatogram of PBS-washed mycelium of *S. commune*. Peak numbers are indicated together with elution times.

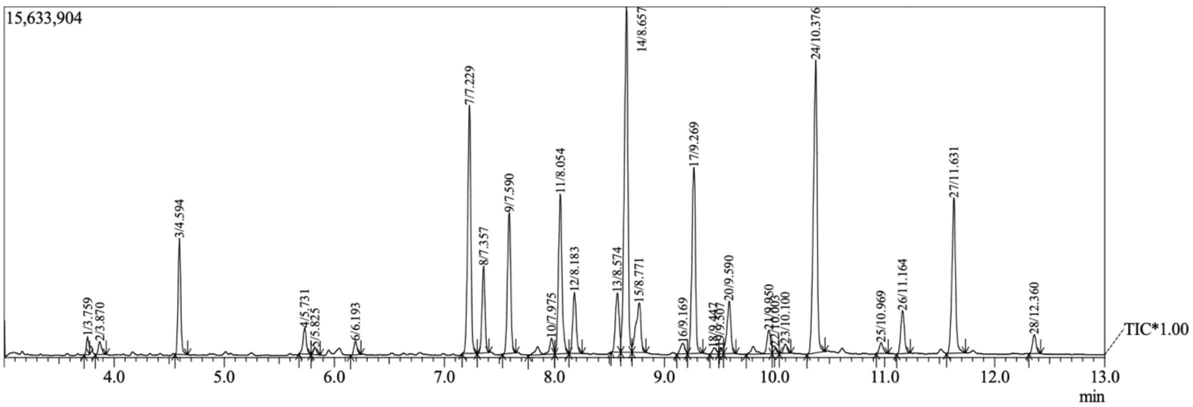

Figure S12. GLC-Chromatogram of SDS-washed mycelium of *S. commune*. Peak numbers are indicated together with elution times.

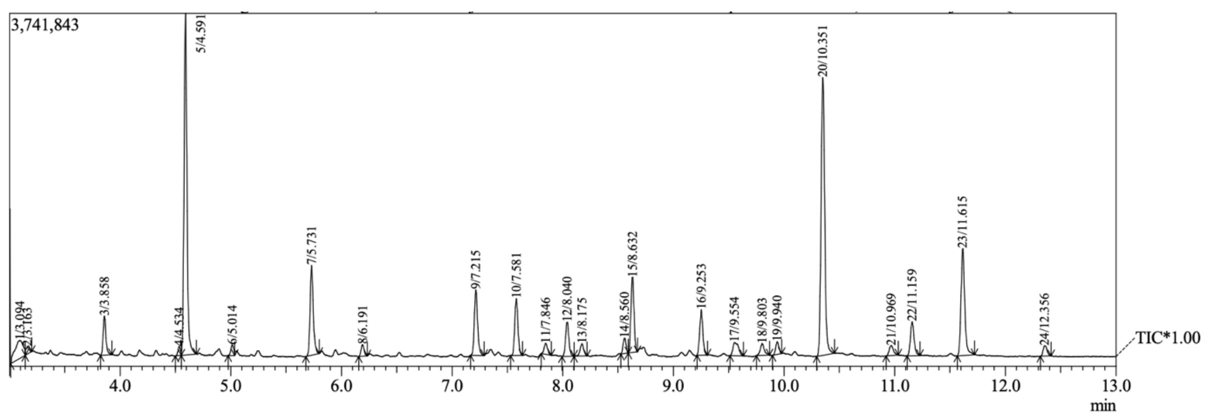

Figure S13. GLC-Chromatogram of alkali treated mycelium of *S. commune*. Peak numbers are indicated together with elution times.

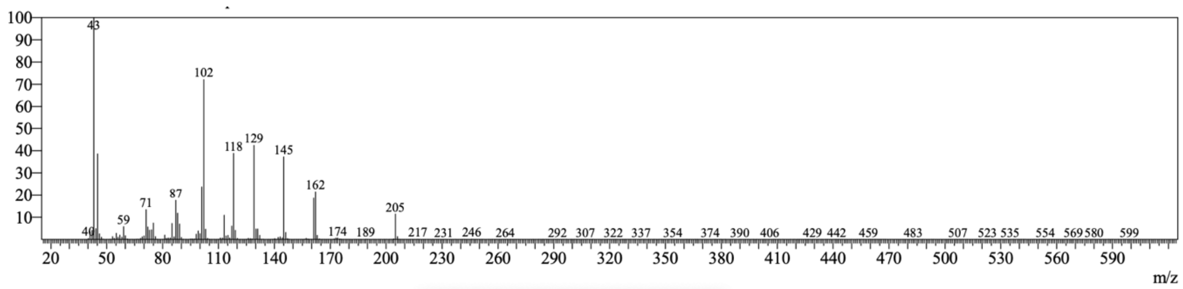

Figure S14. Characteristic fragmentation pattern for a terminal hexose (Hex*p*(1→) taken from the untreated sample.

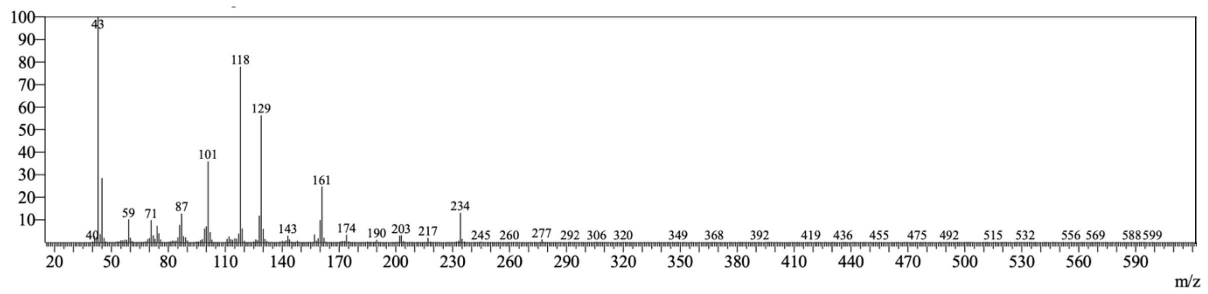

Figure S15. Characteristic fragmentation pattern for a 1,3 linked hexose (→3)Hex*p*(1→) taken from the untreated sample.

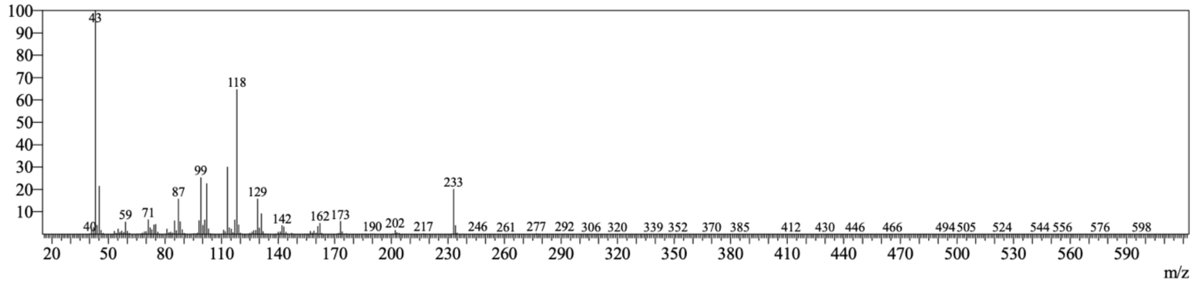

Figure S16. Characteristic fragmentation pattern for a 1,4 linked hexose (→4)Hex*p*(1→) taken from the untreated sample.

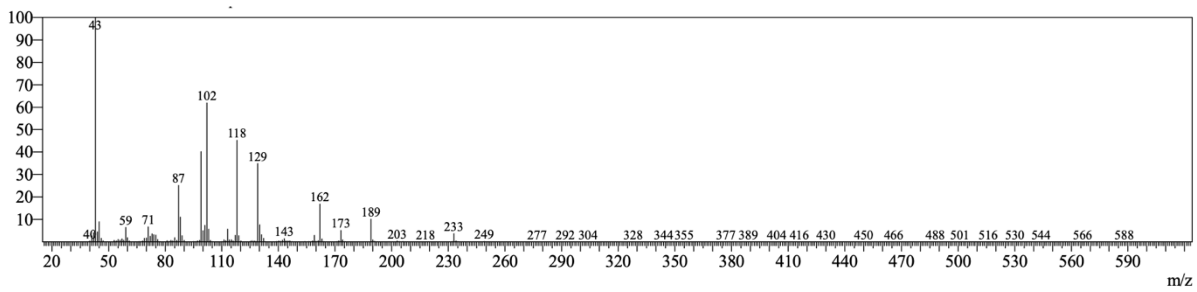

Figure S17. Characteristic fragmentation pattern for a 1,6 linked hexose (→6)Hex*p*(1→) taken from the untreated sample.

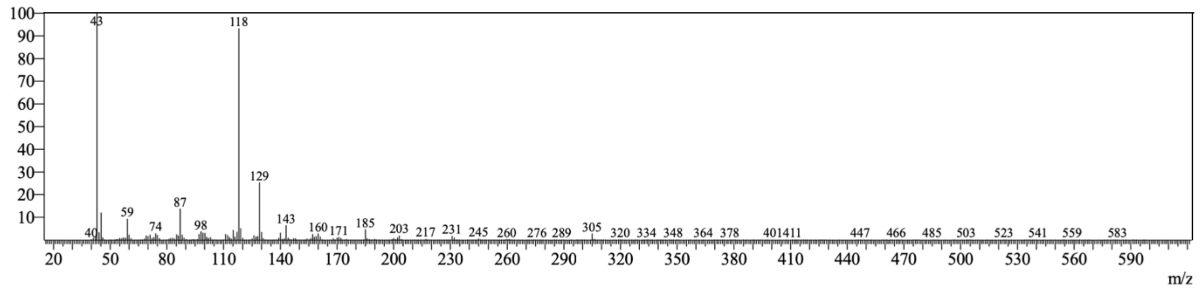

Figure S18. Characteristic fragmentation pattern for a 3,4 linked hexose (→3,4)Hex*p*(1→) taken from the untreated sample.

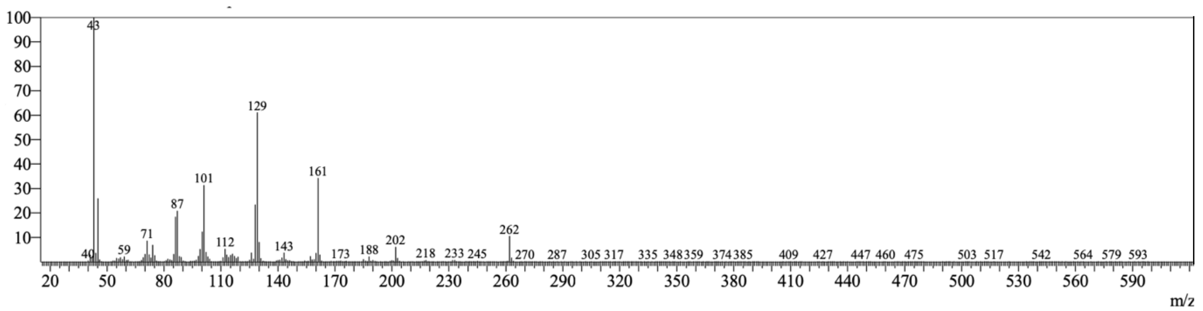

Figure S19. Characteristic fragmentation pattern for a 2,3 linked hexose (→2,3)Hex*p*(1→) taken from the untreated sample.

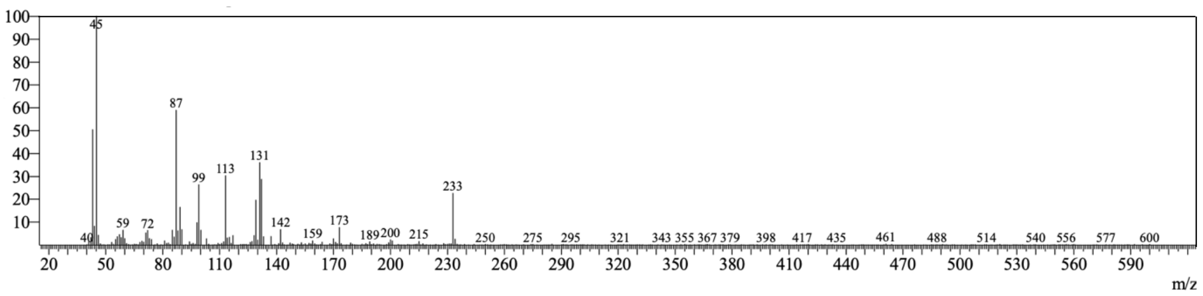

Figure S20. Characteristic fragmentation pattern for a 2,4 linked hexose (→2,4)Hex*p*(1→) taken from the PBS washed sample.

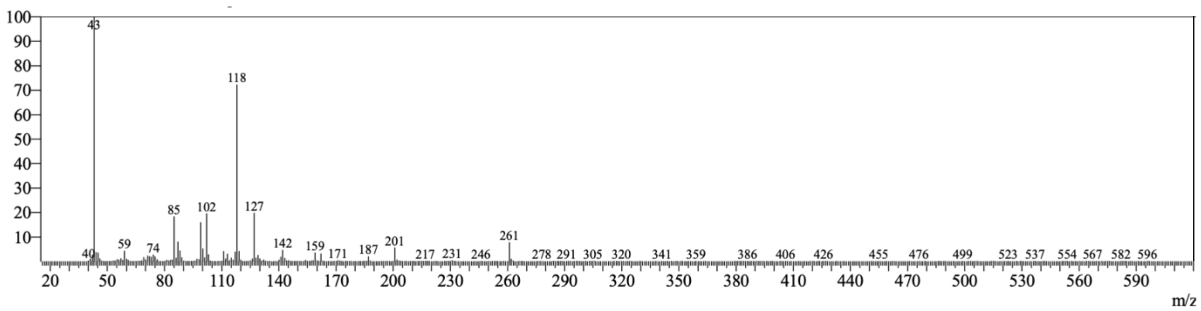

Figure S21. Characteristic fragmentation pattern for a 4,6 linked hexose (→4,6)Hex*p*(1→) taken from the untreated sample.

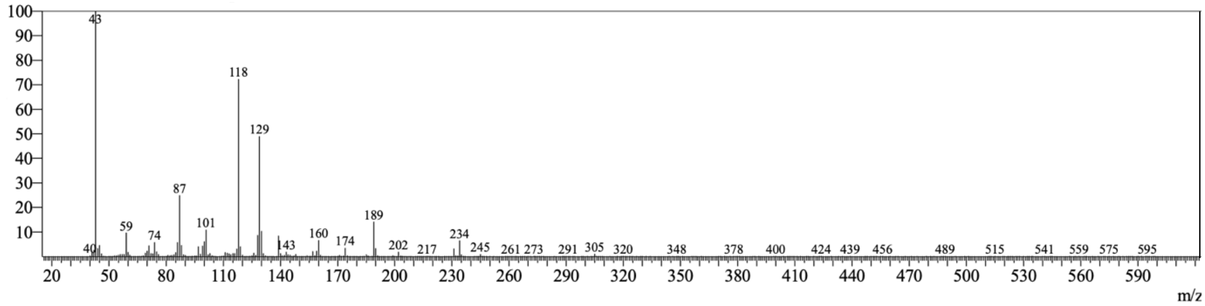

Figure S22. Characteristic fragmentation pattern for a 3,6 linked hexose (→3,6)Hex*p*(1→) taken from the untreated sample.

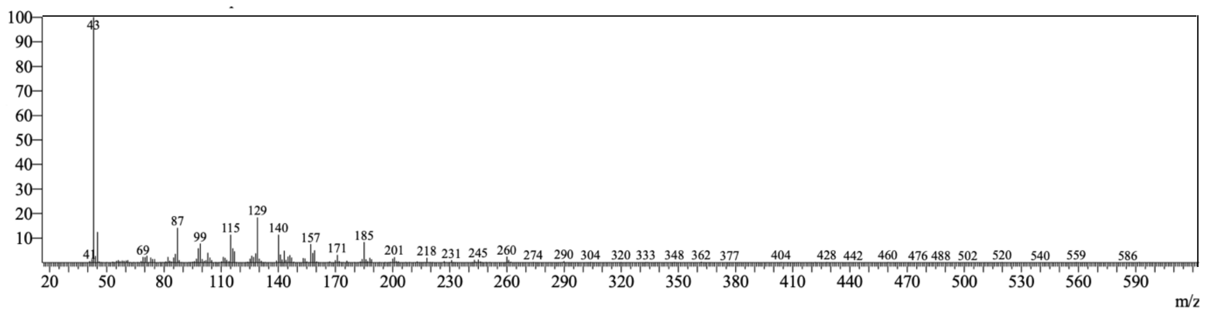

Figure S23. Characteristic fragmentation pattern for a 2,3,4 linked hexose (→2,3,4)Hex*p*(1→) taken from the untreated sample.

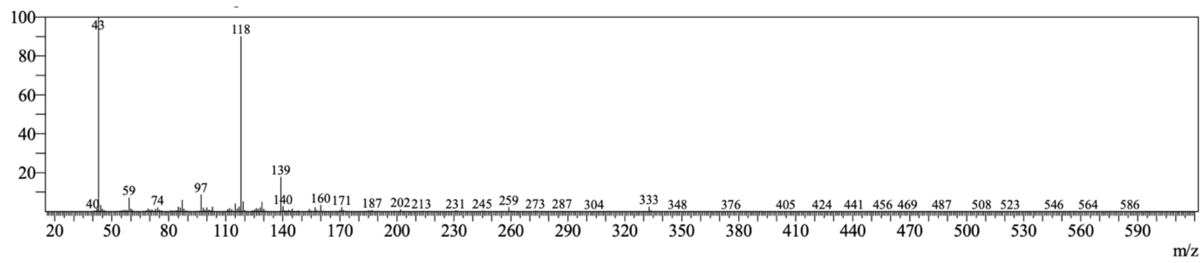

Figure S24. Characteristic fragmentation pattern for a 3,4,6 linked hexose (→3,4,6)Hex*p*(1→) taken from the untreated sample.

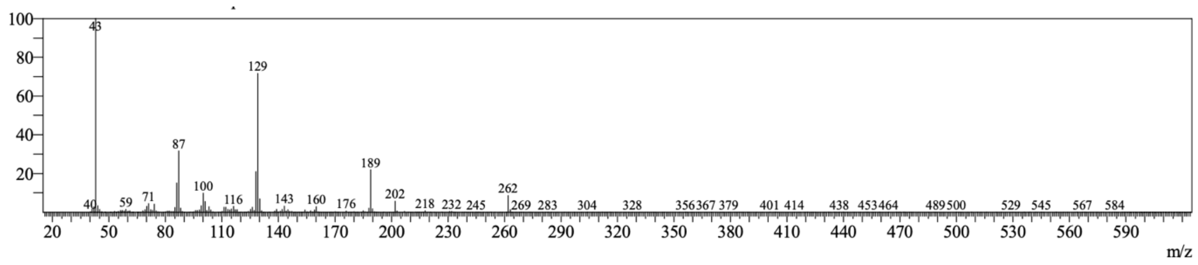

Figure S25. Characteristic fragmentation pattern for a 2,3,6 linked hexose (→2,3,6)Hex*p*(1→) taken from the untreated sample.

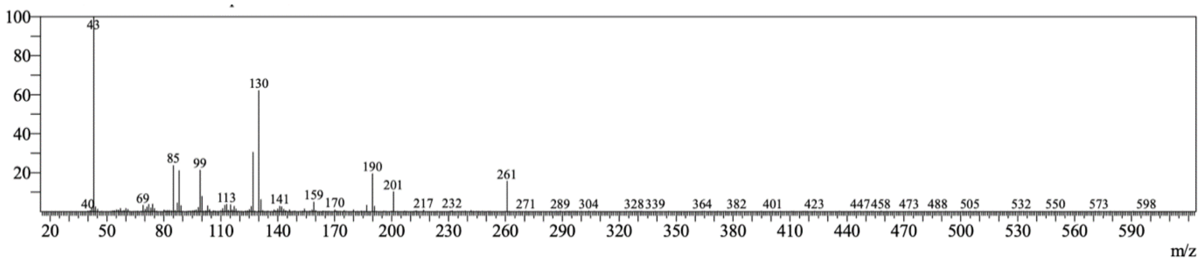

Figure S26. Characteristic fragmentation pattern for a 2,4,6 linked hexose (→2,4,6)Hex*p*(1→) taken from the SDS treated sample.

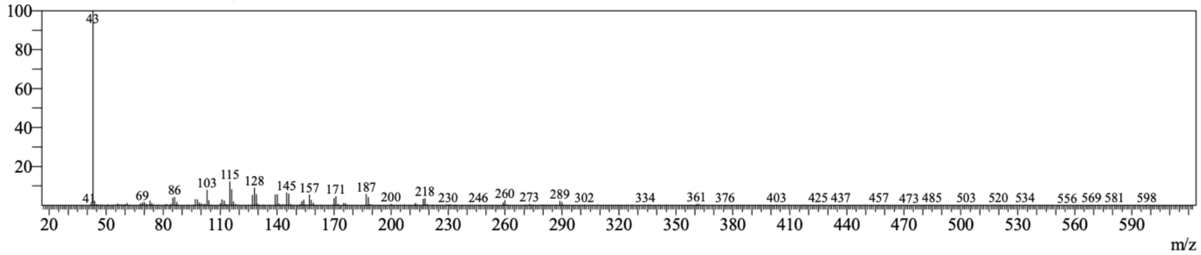

Figure S27. Characteristic fragmentation pattern for a 2,3,4,6 linked hexose (→2,3,4,6)Hex*p*(1→) taken from the untreated sample.

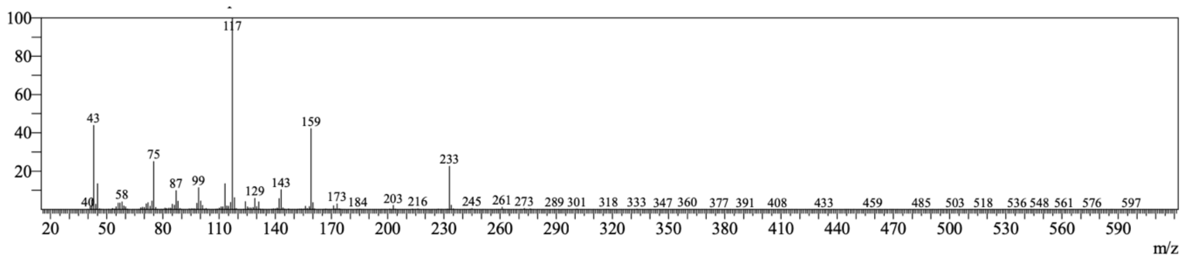

Figure S28. Characteristic fragmentation pattern for a 1,4 linked hexosamine (→4)Hex*p*NAc(1→) taken from the untreated sample.

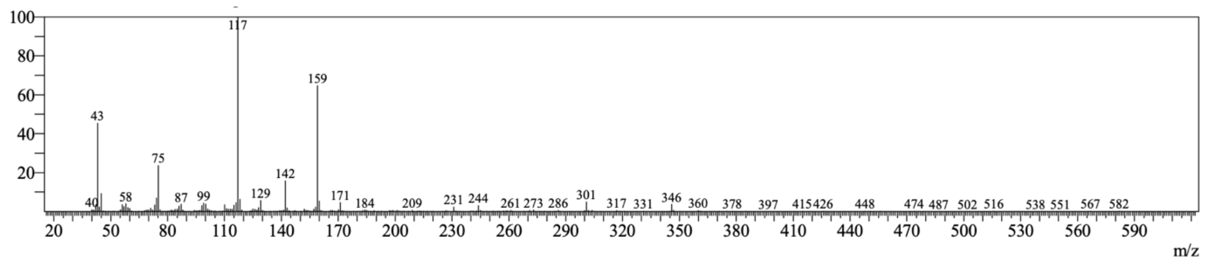

Figure S29. Characteristic fragmentation pattern for a 3,4 linked hexosamine (→3,4)Hex*p*NAc(1→) taken from the alkali treated sample.

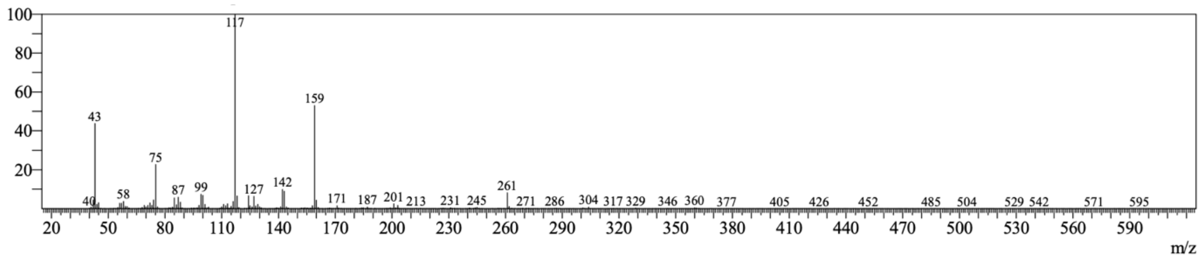

Figure S30. Characteristic fragmentation pattern for a 4,6 linked hexosamine (→4,6)Hex*p*NAc(1→) taken from the alkali treated sample.

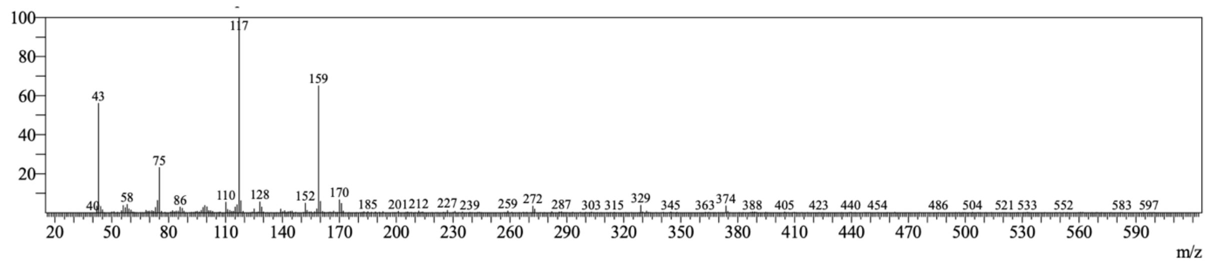

Figure S31. Characteristic fragmentation pattern for a 3,4,6 linked hexosamine (→3,4,6)Hex*p*NAc(1→) taken from the alkali treated sample.

Supplementary References

Alquini, G., Carbonero, E. R., Rosado, F. R., Cosentino, C., & Iacomini, M. (2004). Polysaccharides from the fruit bodies of the basidiomycete Laetiporus sulphureus (Bull.: Fr.) Murr. *FEMS Microbiology Letters*, *230*(1), 47-52.

Andronesi, O. C.; Becker, S.; Seidel, K.; Heise, H.; Young, H. S.; Baldus, M (2005)., Determination of membrane protein structure and dynamics by magic-angle-spinning solid-state NMR spectroscopy. *Journal of the American Chemical Society 127* (37), 12965-12974.

Baldus, M.; Petkova, A. T.; Herzfeld, J.; Griffin, R. G. (1998), Cross polarization in the tilted frame: assignment and spectral simplification in heteronuclear spin systems. *Molecular Physics*, *95* (6), 1197-1207

Baldus, M., & Meier, B. H. (1996). Total Correlation Spectroscopy in the Solid State. The Use of Scalar Couplings to Determine the Through-Bond Connectivity. *Journal of Magnetic Resonance, Series A*, *121*(1), 65–69. https://doi.org/10.1006/JMRA.1996.0137

Bilan, M. I., Grachev, A. A., Shashkov, A. S., Kelly, M., Sanderson, C. J., Nifantiev, N. E., & Usov, A. I. (2010). Further studies on the composition and structure of a fucoidan preparation from the brown alga Saccharina latissima. *Carbohydrate research*, *345*(14), 2038-2047.Bock et al 1983

Black, I., Heiss, C., & Azadi, P. (2019). Comprehensive monosaccharide composition analysis of insoluble polysaccharides by permethylation to produce methyl alditol derivatives for gas chromatography/mass spectrometry. *Analytical chemistry*, *91*(21), 13787-13793.

Chakraborty, I., Mondal, S., Rout, D., & Islam, S. S. (2006). A water-insoluble (1→ 3)-β-d-glucan from the alkaline extract of an edible mushroom Termitomyces eurhizus. *Carbohydrate Research*, *341*(18), 2990-2993.

Chang, Y. W., & Lu, T. J. (2004). Molecular characterization of polysaccharides in hot-water extracts of Ganoderma lucidum fruiting bodies. *Journal of Food and Drug Analysis*, *12*(1), 59-67.

Dong, Q., Yao, J., Yang, X. T., & Fang, J. N. (2002). Structural characterization of a water-soluble β-D-glucan from fruiting bodies of *Agaricus blazei* Murr. *Carbohydrate Research*, *337*(15), 1417-1421.

Fontaine, T., Simenel, C., Dubreucq, G., Adam, O., Delepierre, M., Lemoine, J., ... & Latgé, J. P. (2000). Molecular organization of the alkali-insoluble fraction ofAspergillus fumigatus cell wall. *Journal of Biological Chemistry*, *275*(36), 27594-27607.

Fung, B. M., Khitrin, A. K., & Ermolaev, K. (2000). An Improved Broadband Decoupling Sequence for Liquid Crystals and Solids. *Journal of Magnetic Resonance*, *142*(1), 97–101. https://doi.org/10.1006/JMRE.1999.1896

Gómez-Miranda, B., Prieto, A., Leal, J. A., Ahrazem, O., Jiménez-Barbero, J., & Bernabé, M. (2003). Differences among the cell wall galactomannans from Aspergillus wentii and Chaetosartorya chrysella and that of Aspergillus fumigatus. *Glycoconjugate journal*, *20*(4), 239-246.

Gonzaga, M. L. C., Ricardo, N. M., Heatley, F., & Soares, S. D. A. (2005). Isolation and characterization of polysaccharides from Agaricus blazei Murill. *Carbohydrate Polymers*, *60*(1), 43-49.

Hohwy, M.; Rienstra, C. M.; Jaroniec, C. P.; Griffin, R. G. (1999), Fivefold symmetric homonuclear dipolar recoupling in rotating solids: Application to double quantum spectroscopy. *The Journal of Chemical Physics*, *110* (16), 7983-7992.

Jang, M. K., Kong, B. G., Jeong, Y. I., Lee, C. H., & Nah, J. W. (2004). Physicochemical characterization of α-chitin, β-chitin, and γ-chitin separated from natural resources. *Journal of Polymer Science Part A: Polymer Chemistry*, *42*(14), 3423-3432.

Kameda, T. (2004). Molecular structure of crude beeswax studied by solid-state 13C NMR. *Journal of Insect Science*, *4*(1).

Luo, X., Xu, X., Yu, M., Yang, Z., & Zheng, L. (2008). Characterisation and immunostimulatory activity of an α-(1→ 6)-d-glucan from the cultured Armillariella tabescens mycelia. *Food chemistry*, *111*(2), 357-363.

Marchessault, R. H., Taylor, M. G., & Winter, W. T. (1990). 13C CP/MAS NMR spectra of poly-β-D (1→ 4) mannose: mannan. *Canadian journal of chemistry*, *68*(7), 1192-1195.

McIntyre, D. D., & Vogel, H. J. (1993). Structural studies of pullulan by nuclear magnetic resonance spectroscopy. *Starch-Staerke (Germany)*.

Mondal, S., Chakraborty, I., Pramanik, M., Rout, D., & Islam, S. S. (2004). Structural studies of water-soluble polysaccharides of an edible mushroom, Termitomyces eurhizus. A reinvestigation. *Carbohydrate research*, *339*(6), 1135-1140.

Morris, G. A., & Freeman, R. (1979). Enhancement of nuclear magnetic resonance signals by polarization transfer. *Journal of the American Chemical Society*, *101*(3), 760-762.

Pang, X., Yao, W., Yang, X., Xie, C., Liu, D., Zhang, J., & Gao, X. (2007). Purification, characterization and biological activity on hepatocytes of a polysaccharide from Flammulina velutipes mycelium. *Carbohydrate Polymers*, *70*(3), 291-297.

Petkowicz, C. D. O., Reicher, F., Chanzy, H., Taravel, F. R., & Vuong, R. (2001). Linear mannan in the endosperm of Schizolobium amazonicum. *Carbohydrate Polymers*, *44*(2), 107-112.

Shaka, A. J., Keeler, J., Frenkiel, T., & Freeman, R. A. Y. (1983). An improved sequence for broadband decoupling: WALTZ-16. *Journal of Magnetic Resonance (1969)*, *52*(2), 335-338.

Smiderle, F. R., Olsen, L. M., Carbonero, E. R., Baggio, C. H., Freitas, C. S., Marcon, R., ... & Iacomini, M. (2008). Anti-inflammatory and analgesic properties in a rodent model of a (1→ 3),(1→ 6)-linked β-glucan isolated from Pleurotus pulmonarius. *European journal of pharmacology*, *597*(1-3), 86-91.

Weingarth, M., Bodenhausen, G., & Tekely, P. (2010). Broadband magnetization transfer using moderate radio-frequency fields for NMR with very high static fields and spinning speeds. *Chemical Physics Letters*, *488*(1-3), 10-16.

Yalin, W., Cuirong, S., & Yuanjiang, P. (2006). Studies on isolation and structural features of a polysaccharide from the mycelium of an Chinese edible fungus (Cordyceps sinensis). *Carbohydrate Polymers*, *63*(2), 251
